# Tailoring noble metal nanoparticle designs to enable sensitive lateral flow immunoassay

**DOI:** 10.7150/thno.67184

**Published:** 2022-01-01

**Authors:** Xirui Chen, Lu Ding, Xiaolin Huang, Yonghua Xiong

**Affiliations:** 1 State Key Laboratory of Food Science and Technology, School of Food Science and Technology, Nanchang University, Nanchang 330047, P. R. China.; 2 Hypertension Research Institute of Jiangxi Province, Department of Cardiology, The First Affiliated Hospital of Nanchang University, Nanchang, Jiangxi 330006, P. R. China.; 3 Jiangxi-OAI Joint Research Institute, Nanchang University, Nanchang 330047, P. R. China.

**Keywords:** lateral flow immunoassay, noble metal nanoparticles, nanoparticle design, engineering

## Abstract

Lateral flow immunoassay (LFIA) with gold nanoparticles (AuNPs) as signal reporters is a popular point-of-care diagnostic technique. However, given the weak absorbance of traditional 20-40 nm spherical AuNPs, their sensitivity is low, which greatly limits the wide application of AuNP-based LFIA. With the rapid advances in materials science and nanotechnology, the synthesis of noble metal nanoparticles (NMNPs) has enhanced physicochemical properties such as optical, plasmonic, catalytic, and multifunctional activity by simply engineering their physical parameters, including the size, shape, composition, and external structure. Using these engineered NMNPs as an alternative to traditional AuNPs, the sensitivity of LFIA has been significantly improved, thereby greatly expanding the working range and application scenarios of LFIA, particularly in trace analysis. Therefore, in this review, we will focus on the design of engineered NMNPs and their demonstration in improving LFIA. We highlight the strategies available for tailoring NMNP designs, the effect of NMNP engineering on their performance, and the working principle of each engineering design for enhancing LFIA. Finally, current challenges and future improvements in this field are briefly discussed.

## 1. Introduction

Lateral flow immunoassay (LFIA) is a popular point-of-care testing technique because it meets the ASSURED criteria of the WHO 1. It provides a wide range of applications in food safety, environmental monitoring, and clinical diagnosis 2, 3. For example, LFIA has played a crucial role in the response and management of coronavirus disease 2019 4, particularly in resource-limited areas with low access to the real-time reverse transcriptase quantitative polymerase chain reaction testing 5. In a typical LFIA test, effective detection of the analyte depends on the specific immunological recognition of analytes and detection-antibody-conjugated nanoparticle labels and the accumulation of nanoparticle labels at the test and control zones by the capillary force, thereby achieving effective signal transduction 6. In sandwich LFIA testing, the label capture is positively related to the analyte concentration; as a result, the higher the concentration of the analyte, the stronger the signal on the test line. On the contrary, for competitive LFIA testing, the signal strength of the nanoparticle label is negatively correlated with the analyte concentration. Over the past few decades, spherical gold nanoparticles (AuNPs) have become a major component of nanoparticle labels in LFIA because of their visual readout without any instruments, good colloidal stability, and easy functionalization 7. However, traditional LFIA, which uses 20-40 nm AuNPs as signal labels, has suboptimal sensitivity compared with other immunoassay methods, thereby greatly limiting its wide applications in the detection of trace analytes. Therefore, developing strategies to improve the sensitivity and extend the application range of the LFIA is necessary 8.

In recent years, a myriad of approaches has been enabled to enhance the LFIA sensitivity. These strategies primarily involve the introduction of sample treatments to preconcentrate the analyte, the screening of high-affinity recognition elements 9, the development of novel nanoparticle labels and signal transduction techniques with increased sensitivity 8, and the combination of various signal amplification methods 6. With these improvements, the detection performance of LFIA in sensitivity has been significantly enhanced. Recently, several pivotal review papers have discussed the progress and achievements in reducing the limit of detection (LOD) and improving the sensitivity of LFIA. Nevertheless, these improvements could result in increased cost and complexity and compromised portability, which is contrary to the main technical strength of LFIA. Consequently, traditional colorimetric signal transduction with AuNPs as labels still dominates the LFIA because of its superiorities over other alternative labels and signal readout patterns.

As mentioned earlier, the generation of detectable colorimetric signals relies on the adequate accumulation of AuNP labels in the detected region. Therefore, the low sensitivity of traditional colorimetric LFIA can be effectively solved by enhancing the signal strength of the label 6. Noble metal nanoparticles (NMNPs), including AuNPs, are a new functional nanomaterial, which have received considerable attention in the field of bioanalysis because of their unique physical and chemical properties, including optical 10, 11, plasmonic 12, and catalytic activities 13. NMNPs not only serve as traditional colorimetric labels, but also achieve other signal transduction principles and functions, such as surface-enhanced Raman scattering (SERS) 14, fluorescence 15-19, and enzyme mimicking 20, 21, thereby contributing to highly sensitive detection in LFIA 22-25. Furthermore, the great success in large-scale synthesis of high-quality NMNPs has promoted their applications for the revolution of LFIA technologies. In particular, the physicochemical properties of NMNPs are reported to be closely associated with their size, shape, elemental composition, external structure, interparticle distance, and dielectric constant, thereby allowing us to tailor the properties of NMNPs through controlling these physical parameters 20, 26, 27. For example, localized surface plasmon resonance (LSPR), a representative optical feature of NMNPs, can be selectively tuned by simply engineering the nanoparticle size 28, shape 29, composition 30, and construction 31. In general, the LSPR absorption of NMNPs gradually increases with the increase of nanoparticle size, thereby enabling more sensitive colorimetric signal output when larger-sized NMNPs are used as LFIA labels 32, 33. Moreover, compared with spherical nanoparticles of similar size, anisotropic NMNPs with more complex structures display stronger optical signal sensitivity, which is favorable for enhanced detection sensitivity in LFIA 34, 35. The enzyme-mimicking activities of NMNPs (*e.g.*, AuNPs or AgNPs) can be significantly enhanced by incorporating with platinum-group metals, which can endow them excellent peroxidase-like activities for amplifying the colorimetric signal intensity generated from the label by additional enzymatic deposition step, thereby improving LFIA sensitivity 36. In addition, NMNPs can be equipped with new functions by compositing other functional materials, thereby facilitating their multifunctional uses in LFIA 37-39. Collectively, manipulating the properties of NMNPs by controlling one of the parameters or a combination of them is an effective strategy to achieve the best performance of NMNPs in LFIA.

**Scheme 1:**
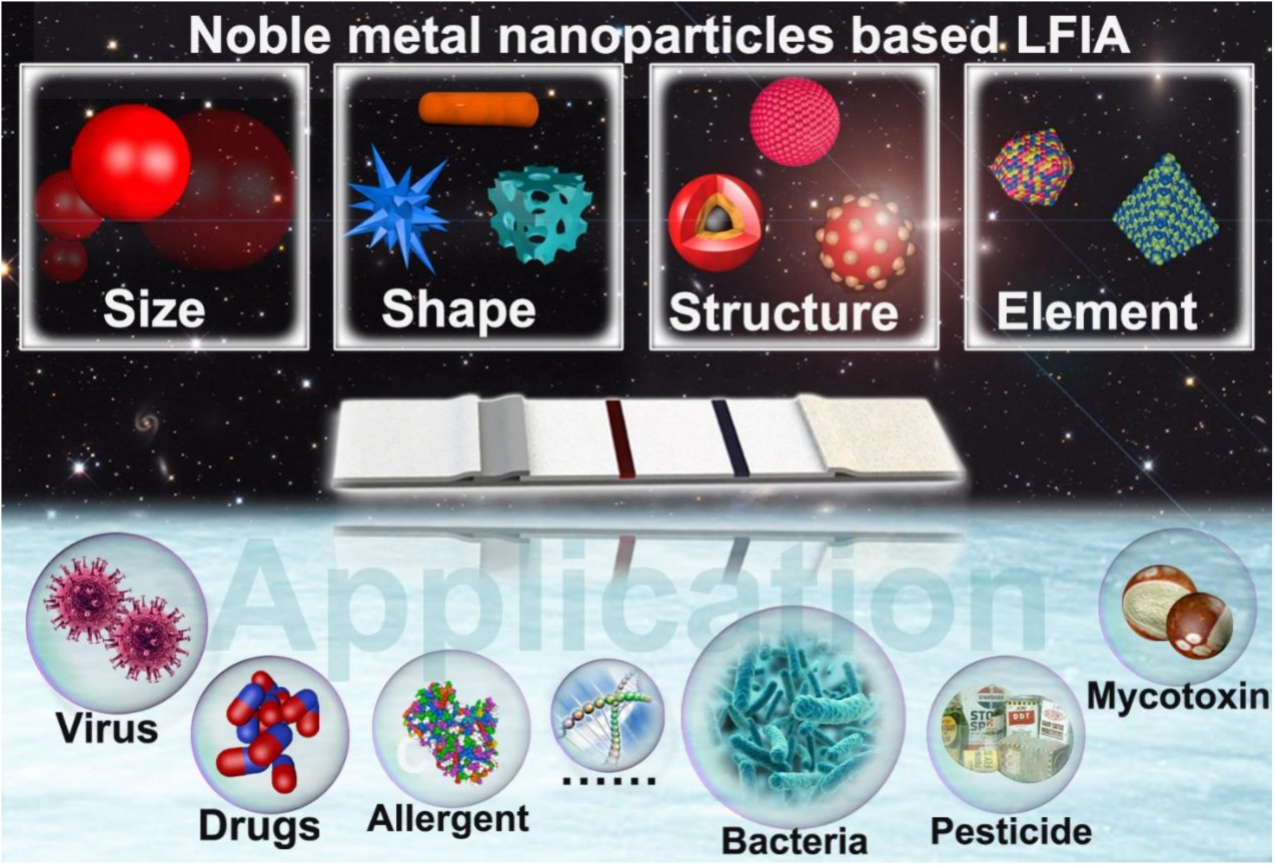
Schematic illustration of engineered noble-metal nanoparticles and their application in LFIA.

With this design concept in mind, numerous excellent studies on the fine tuning of the physical parameters of NMNPs and their uses in enhancing the performance of LFIA have been reported 40-42. The past few decades have witnessed great progress and achievements in tailoring NMNP designs to improve LFIA. Although several publications have been conducted on the principles, labels, sensitivities, multiplexing abilities, and applications of LFIA 43-48, to our knowledge, no systematic and comprehensive review has been conducted on the design of engineered NMNPs and their role in enhancing the LFIA performance. Herein, in this contribution, we focus on exploiting NMNPs by engineering their physical parameters, including size, shape, composition, and external structures, to improve LFIA (**Scheme 1**). We highlight the available strategies to tailor the NMNP designs, the impacts of NMNP engineering on their properties, and the underlying enhancement mechanisms of each nanoparticle engineering design in LFIA applications. This review will conclude with a discussion of existing challenges and further improvements.

## 2. Nanoparticle size

The optical properties of NMNPs are closely related to their sizes. The relationship between them has been extensively studied 49. In general, large NMNPs have strong optical signal transduction relative to small ones because the molar extinction coefficients of NMNPs increase with the increase of the nanoparticle size 50, 51. For instance, 80 nm AuNPs show about two orders of magnitude increase in the molar extinction coefficient compared with 15 nm AuNPs 52. Therefore, large NMNPs with high optical intensity contribute to the sensitivity of LFIA theoretically. However, some studies have indicated that the use of oversized nanoparticles as LFIA labels can reduce the sensitivity 33, 53. Thus, investigating the role of the size of NMNPs on their performance in LFIA is of great significance. The nanoparticle size not only affects the signal transduction but also impacts the interaction behavior of the nanoparticles with biomolecules or other components involved in LFIA. In addition, metal *in situ* growth (MISG), as a commonly used signal amplification strategy, has been widely proposed to enhance the sensitivity of AuNP-LFIA by increasing the size of AuNP and the optical signal strength 54, 55. In this content, gold 56-58, silver 59, 60, and copper 61, 62 growth strategies are currently the potential approaches for amplifying the optical signal intensity of AuNPs by reducing metal ions into metallic elements to deposit on the surface of AuNPs, followed by a significant increase in the nanoparticle size. With these engineering designs of nanoparticle size, the optical signal transduction of nanoparticle labels is remarkably enhanced, thereby improving the LFIA sensitivity and attracting enormous interests for applications in bioanalysis, particularly in trace analysis. Herein, we will focus on the currently available strategies to precisely control the size of NMNPs and the latest advancement of applying size-dependent nanoparticle reporters to enhance the LFIA performance (**Table 1**). Moreover, the MISG-based signal amplification technologies and their uses in enhancing the LFIA sensitivity are discussed (**Table 2**).

### 2.1. Strategies for nanoparticle size regulation

**Figure 1:**
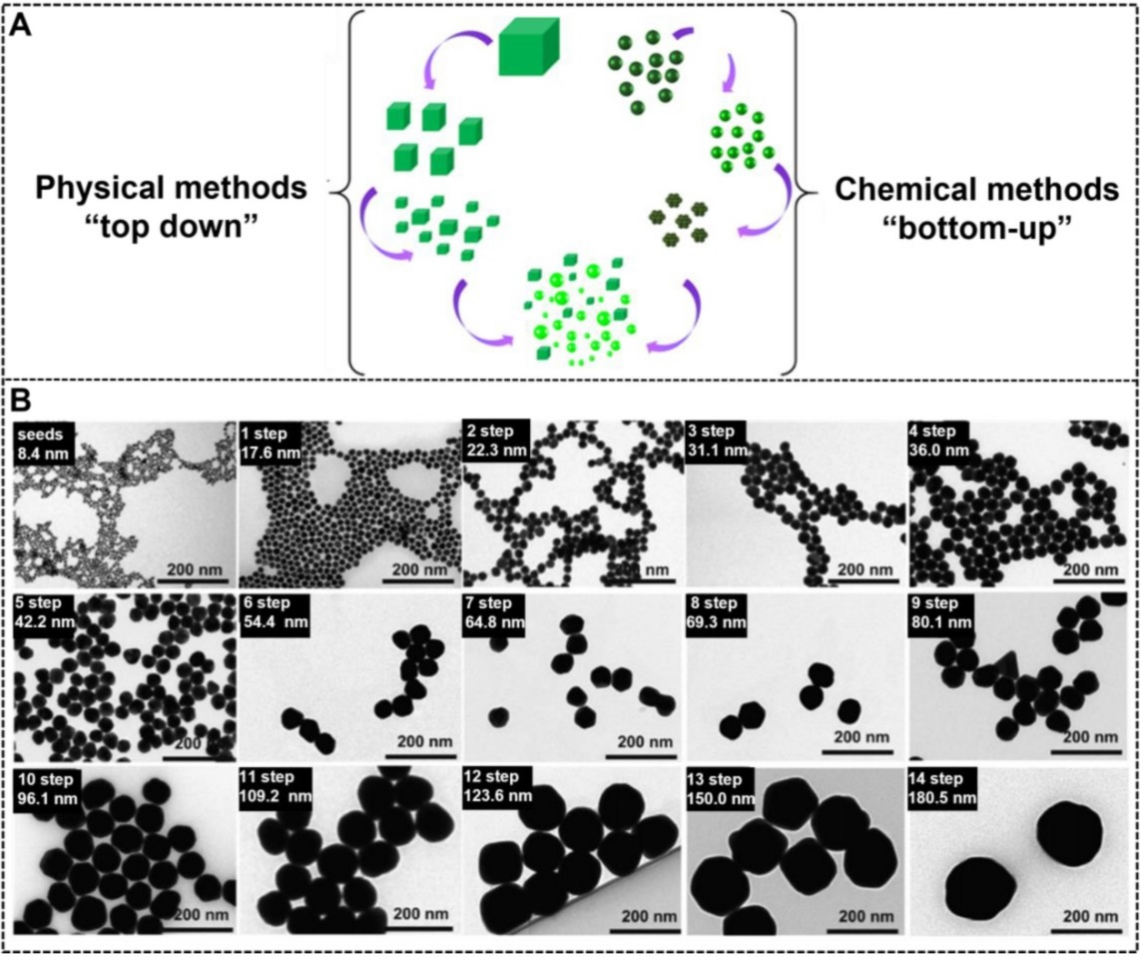
**(A)** Schematic illustration of synthesizing metal nanoparticles with different sizes by physical and chemical methods. Adapted with permission from 20. Copyright 2019 Springer Nature. **(B)** Transmission electron microscopy images of AuNPs with the size increases from 8.4 ± 1.0 to 180.5 ± 10.7 nm obtained by seeded growth. Adapted with permission from 78. Copyright 2011 American Chemical Society.

The synthesis of NMNPs with a variety of sizes can be achieved in the solid or liquid state by using two classical approaches, namely, “top-down'' and ''bottom-up'' (**Figure 1A**) 20, 63. The top-down method begins with a macroscopic bulk metal material entity and achieves structural dimensions in the nanoscale by using the physical and lithographic technologies, such as micropatterning 64, mechanical grinding 65, pyrolysis 66, mechanical alloying 67, and laser ablation 68, 69. However, obtaining high-quality NMNPs with controlled size and nanocrystal using these technologies is difficult. By contrast, the bottom-up approach usually uses ionic, atomic, or molecular units to assemble and form the nano-sized structures through diverse processes 70. From the generation of the components to their growth and assembly into nanoentities, the bottom-up method is primarily regulated on the basis of the chemical synthesis mechanism 71. Compared with the top-down method, the bottom-up approach provides more possibilities for the design and synthesis of nanoparticles with desired size and morphology. Moreover, this method allows the investigation of the structural formation dynamics and structure-property relationship at the atomic or molecular level 72. To date, the currently available bottom-up methods involve the Turkevich, Brust-Schiffrin, microemulsion, electrochemical, photochemical, microwave, green synthesis, and biosynthesis methods 73. Although these methods have gained huge success in synthesizing various size-tunable NMNPs, the size range of NMNPs larger than 100 nm via such methods remains a challenge. The seed-mediated growth method based on the nucleation and growth processes has attracted great interest and has become the potential and versatile method to control the size of NMNPs 74, 75. In general, the classic seed-mediated growth process involves two steps: the synthesis of seed nanoparticles and their subsequent growth in a growth solution consisting of metal precursors, reducing agents, and structure-directing agents (SDA). Unlike the above-mentioned methods, the seed-mediated growth method exhibits more advantages because it can allow the structural design by the selection of seed nanoparticles and the manipulation of crystal growth conditions. In this case, the seed crystals are step-wise enlarged by adding the metal atoms; thus, the size of nanoparticles can be readily controlled by varying the synthetic conditions, including the seeds, physical parameters (e.g., pH and temperature), metal precursors, reducing agents, or additives and impurities 76-78. Taking the synthesis of AuNPs as an example, Natan et al. pioneered the seed-mediated growth method to prepare AuNPs of up to 100 nm with improved monodispersity 79. Subsequently, Murphy et al. and Liz-Marzán et al. further improved the seed-mediated growth process to ensure the controlled synthesis of nanoparticles 70. Inspired by these works, a series of kinetically controlled seed-mediated growth strategies was recently reported for synthesizing monodisperse AuNPs with sizes of more than 100 nm by adjusting the reaction conditions. Using this principle, Bastús et al. reported the synthesis of monodisperse citrate-stabilized quasi-spherical AuNPs with the size of up to ~200 nm by regulating the synthetic conditions, including the gold precursor to seed nanoparticle concentration, reaction temperature, pH value, and reducing agents, to enable the dynamic control of growth processes 78. **Figure 1B** shows that the size of AuNPs increases with the increase of the growth step number, and AuNPs of up to ~180.5 ± 10.7 nm in size with a narrow distribution are synthesized after the 14-step growth. Similarly, Xia's group explored a controlled synthesis system for controllable preparation of spherical AuNPs with a size range of 5-150 nm and silver nanocubes with a size range of 30-200 nm by using a successive, seed-mediated growth strategy 76-77.

### 2.2. Effect of nanoparticle size on optical properties

**Figure 2:**
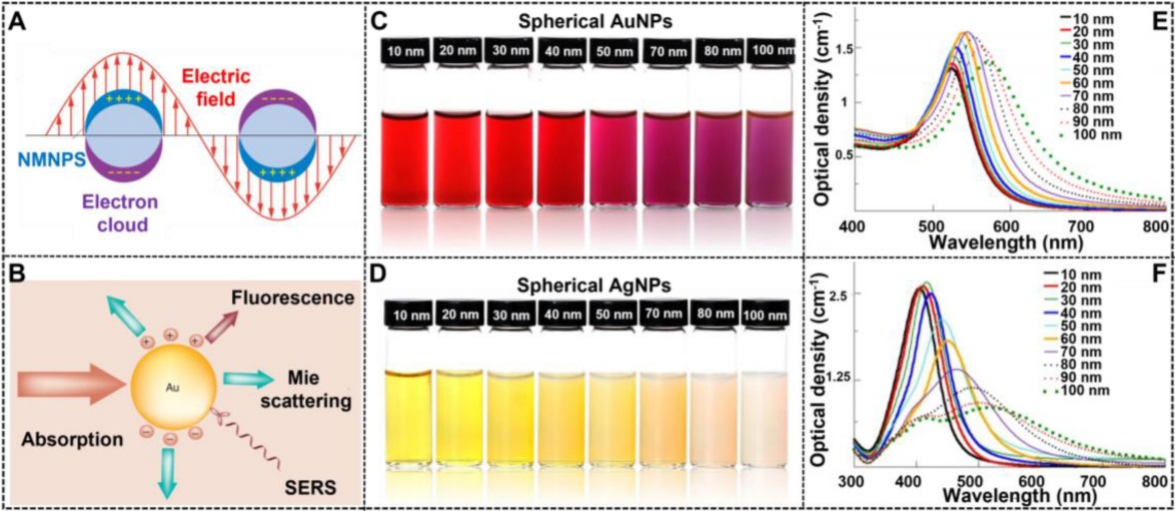
** (A)** Depiction of LSPR of spherical NMNPs. Adapted with permission from 80. Copyright 2009 Annual Reviews. **(B)** Important optical performance caused by the interaction of light with AuNPs, including light absorption, Mie scattering, surface-enhanced luminescence and surface-enhanced Raman scattering from absorbed molecules. Adapted with permission from 81. Copyright 2007 Future Medicine Ltd ISSN. The photographs of AuNPs **(C)** and AgNPs **(D)**. UV-vis spectra of AuNPs **(E)** and AgNPs **(F)**. Adapted with permission from 104. Copyright Fortis Life Sciences Company.

NMNPs have obtained a wide range of applications in various fields because of their unique optical properties, which primarily derive from the LSPR. As shown in **Figure 2A**, LSRP is the coherent oscillation of the free electron away from the equilibrium position when NMNPs are excited by the incident light 80. As a result of the LSPR phenomenon, NMNPs can absorb and/or scatter light at specific wavelengths (**Figure 2B**) 81, thereby giving their solution a certain color. In addition, the LSPR peak position of NMNPs and their solution color can be easily tuned over a wide range of wavelengths and colors through manipulating the size of NMNPs. Using the theoretical calculations and experimental observations, a large number of studies have demonstrated the enhanced optical features of NMNPs with the increase of the size of nanoparticles 49, 52, such as the finely tunable extinction spectra from the ultraviolet regions to the visible regions and even to the near-infrared regions, and the increased extinction cross-section with the molar extinction coefficient ranging from 10^8^ to 10^11^ M^-1^ cm^-1^ 82. For example, the spherical AuNPs with sizes of 30 nm exhibit the maximal LSPR peak at ~525 nm, thereby giving their solution a typical wine-red color. When the size of spherical AuNPs increases up to 100 nm, the maximal LSPR peak is red-shifted to ~560 nm with a brick-red color (**Figures 2C and E**) 104. In addition, spherical AgNPs also display size-dependent variations in the LSPR and color. For example, when the size of silver nanospheres is 50 nm, their major LSPR peak is located at ~420 nm with a distinctive yellow color solution, whereas the LSPR and color of spherical AgNPs will shift to ~485 nm with a characteristic Khaki grey color solution as the size of nanospheres increases up to 100 nm (**Figures 2D and F**) 104. The unique superiority of NMNPs with size-dependent variations in the optical properties allows the customized design and preparation of various high-performance colorimetric signal probes based on the requirements for various applications. Furthermore, these properties provide the potential opportunities for elucidating the relationship between the nanoparticle size and its performances in LFIA.

Noble metal nanoclusters (NMNCs) are the transition between single metal atoms and metal nanoparticles. When the size of metal nanoparticles is down to below 2 nm, consisting of several to dozens of atoms, they exhibit unique photoluminescent properties, thus providing great potential as fluorescence labels for applications in diverse fields 23. However, compared with quantum dots and organic fluorophores, NMNCs show obvious drawbacks for low quantum yield, thus limiting its application in high-sensitivity detection. In recent years, attempts have been devoted to exploring strategies for improving the NMNC brightness. Many promising approaches, such as the modification of fluorescence behaviors (*e.g.,* aggregation induced emission or confined fluorescence enhancement), the optimization of synthesis process (*e.g.,* microwave assisted-homogeneous heating), and the improvement of environmental conditions (*e.g.,* temperature, pressure, and pH), have been developed for preparing highly luminescent nanoclusters. By virtue of these improvements, the synthesis of highly fluorescent NMNCs with the advantages over other fluorescent materials has been successfully achieved.

### 2.3. Size engineering in LFIA

The size of nanoparticles is an important factor to determine their performance in LFIA. Large nanoparticles usually possess stronger optical signal intensities than the small ones, thereby contributing to the improvement of the sensitivity of traditional LFIA theoretically. Therefore, we will describe the latest advancement in size engineering of nanoparticles and their applications in improving LFIA sensitivity.

#### Size effect

**Figure 3:**
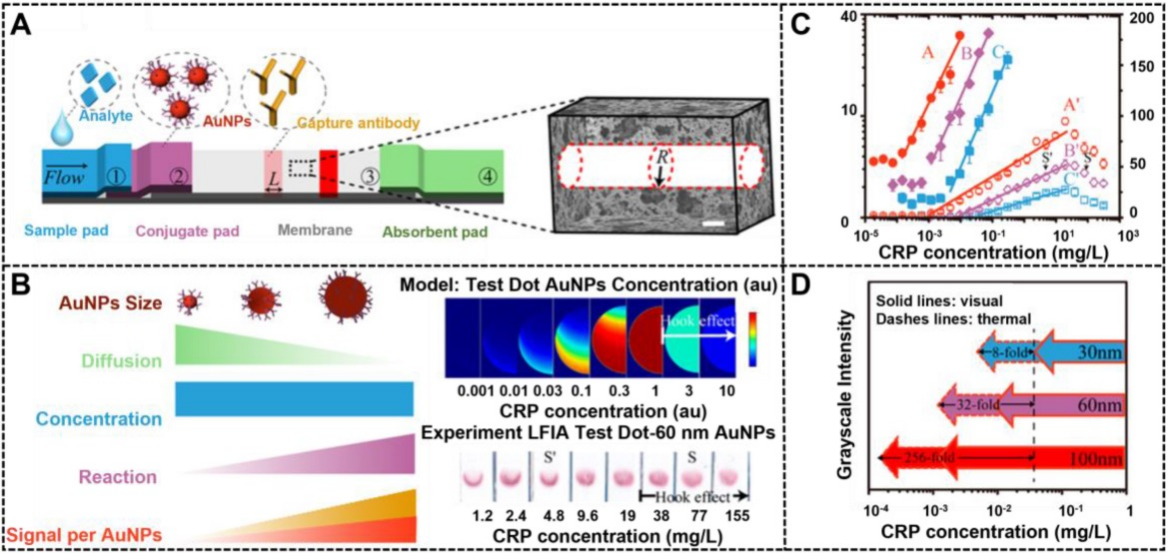
Scaling analysis of the effect of AuNPs size on LFIA. **(A)** Architecture of LFIA, the nitrocellulose membrane is conceptually simplified as bundles of cylindrical pores with radius R. **(B)** Comparison of different sized AuNPs, indicates 100 nm AuNPs can improve LFIA sensitivity due to higher reaction rate and signal per AuNPs. **(C)** COMSOL modeling result of 100 nm AuNP and experimental result of 60nm AuNPs in the test zone of sandwich LFIA of CRP. **(D)** Experimental thermal (solid symbols) and visual (hollow symbols) signals of CRP LFIAs. Adapted with permission from 33. Copyright 2017 American Chemical Society.

The size of nanoparticles plays a decisive role in fabricating high-performance LFIA because the size affects not only signal transduction, but also immunoreaction efficiency between the nanoparticle probe and other components in LFIA. Thanks to the large advances in the synthesis of highly luminescent nanoclusters, NMNCs have been proposed as promising fluorescent labels for improving the LFIA. Jiang et al. first described the successful preparation of highly luminescent green emitting Au nanoclusters (AuNCs) and demonstrated their applications in improving the multiplexed LFIA detection 23. By combining a two-line design and a portable fluorescent reader, the developed multiplex LFIA can allow the simultaneous and quantitative detection of clenbuterol and ractopamine with LODs of 0.003 and 0.023 μg L^-1^, respectively. Nevertheless, the application of NMNCs in LFIA is still limited. When the nanoparticle size exceeds 5 nm, noble metal nanomaterials can effectively generate colorimetric signals by absorbing or scattering incident light and produce thermal contrast signals by the photothermal effect 83. In addition, the intensities of colorimetric or thermal signal linearly increases with the increase of the nanoparticle size, which suggest that the use of large NMNPs as LFIA labels is suitable for highly sensitive LFIA detection. However, the excessive size of nanoparticle labels will have a negative impact on their interaction with biomolecules involved in LFIA, thereby resulting in reduced sensitivity. Therefore, illuminating the effects of the label size and signal intensity on the LFIA performance is desired. In this regard, considerable literature has studied the relationship between the nanoparticle size and LFIA sensitivity 32, 84. For example, Laitinen et al. explored the impact of size of spherical AuNPs in the range of 20 nm to 39 nm on the sensitivity of competitive LFIA 85. Safenkova and coworkers used five kinds of spherical AuNPs with the size ranging from 6.4 nm to 52 nm to investigate the impact of size on the sandwich LFIA platform 86. All these studies confirm that large AuNPs could increase the LFIA sensitivity compared with small AuNPs. However, given the synthetic limitations of large AuNPs, the size range of AuNPs used in these studies is quite narrow, making it difficult to accurately describe the effect of size on LFIA sensitivity. Given the breakthrough of the synthesis of large AuNPs, our group synthesized four kinds of spherical AuNPs with sizes of 20, 60, 100, and 180 nm by a kinetically controlled seed-mediated growth strategy and then applied them as signal reporters in a competitive LFIA to explore the impact of spherical AuNP on the detection sensitivity 53. Using ochratoxin A as a model analyte, the results revealed that spherical AuNPs with an appropriate size of 100 nm could remarkably enhance the signal transduction of LFIA to increase sensitivity because of their increased molar extinction coefficient and binding affinity to target analytes. Under the circumstance, increasing the AuNP size can improve the sensitivity of competitive LFIA. Notably, with the further increase of the AuNP size to 180 nm, the gravity of nanoparticles will dominate their move in the pores of the strip. The reduced Brownian motion of probes at the strip will increase the nonspecific binding of the probe and result in an increase in background signal, thereby reducing the LFIA sensitivity despite the exceptional molar extinction coefficient of 180 nm AuNPs. In addition, when the size of AuNPs exceeds 80 nm, light scattering of spherical AuNPs increases sharply, which disapprove the absorption-dominated signal transduction in LFIA 82. Recently, a similar observation was reported by Zhan et al., who investigated such impacts in sandwich LFIA from the theoretical and experimental perspective 33. Prior to experimental study, COMSOL computational modeling was developed to extend scaling analysis and predict LFIA performance. This model can be used to understand the greater impact of the reaction and convection on AuNP capture than diffusion and to confirm the key parameters that determine the LFIA performance, including AuNP size and concentration, reaction rate constant, and flow rate. With C-reactive protein (CRP) as the model analyte, the modeling results of a sandwich LFIA for CRP are depicted in **Figure 3**, which agree well with the theoretical simulation-assisted experimental results. In their work, 100 nm AuNPs display the largest enhancement in sensitivity in comparison with conventional 30 nm AuNPs. The modeling results demonstrate that large AuNPs (i.e., 400 nm) would reduce the LFIA sensitivity and increase the background because of their settlement on the membrane and slow diffusion rate. This finding is consistent with our results. Thus, we believe that appropriate nanoparticle labeling design contributes to the LFIA performance with higher sensitivity and negligible background.

#### Growth-mediated size increase

**Figure 4:**
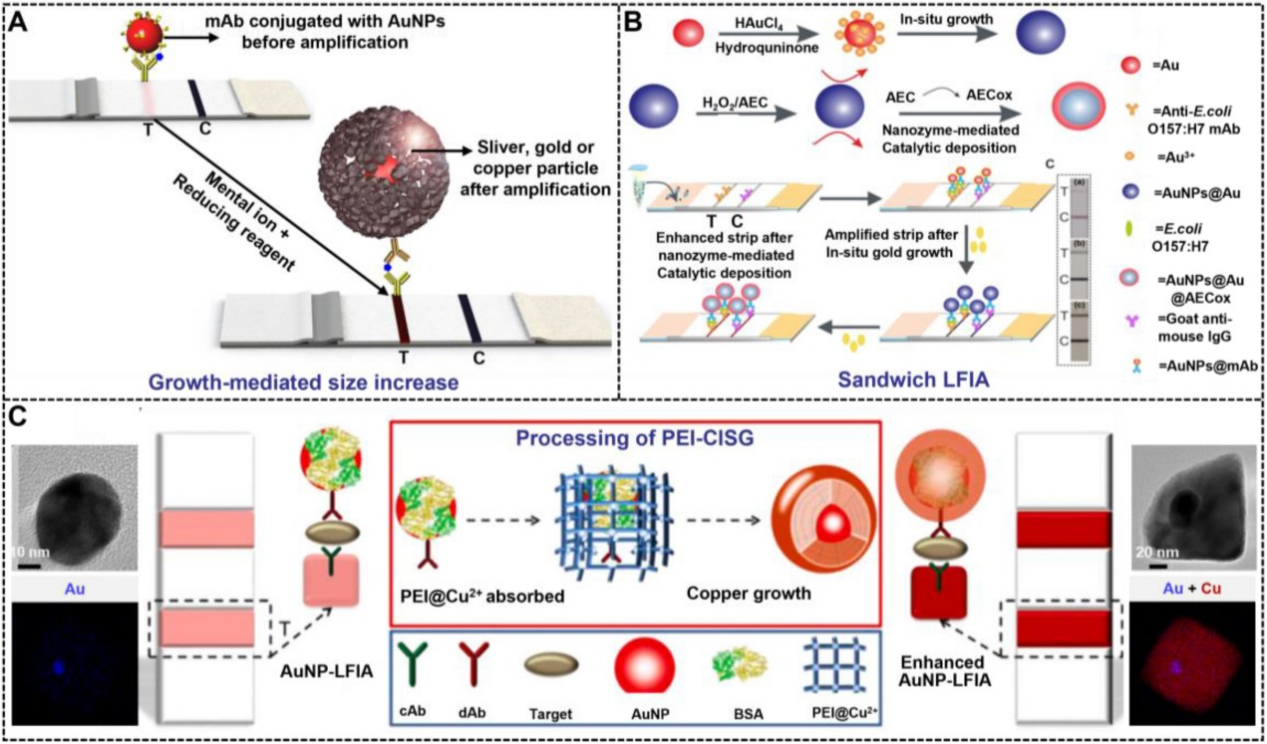
** (A)** The principle of the metal *in-situ* growth-mediated signal amplification in LFIA. **(B)** Schematic of the cascade signal amplification strategy based on the AuNP-LFIA platform for ultrasensitive detection of *E. coli* O157:H7 in milk. Adapted with permission from 58. Copyright 2020 American Chemical Society.** (C)** Schematic illustration of the principle and process of PEI-CISG technology for enhancing AuNP-LFIA. Adapted with permission from 62. Copyright 2021 Elsevier.

The use of large AuNPs with enhanced signal intensity can improve the LFIA sensitivity. However, the increase in sensitivity is relativity limited by the inherent contradictions of size-dependent signal intensity and immunoreaction efficiency. Thus, signal amplification techniques are integrated with conventional AuNP-based LFIA to retain the capture and diffusion of AuNPs on the membrane. This combination does not change the travel of AuNPs on the membrane, and it maximizes their capture. MISG-mediated signal amplification technologies have obtained extensive application in LFIA because of their ultrahigh signal amplification efficiency. Metal growth intensification depends on the reduction of metal ions into metal element to deposit onto the surface of AuNPs, thereby increasing the AuNP size to enhance its colorimetric signal strength and improve the sensitivity of LFIA (**Figure 4A**). Using this amplification strategy, the LOD of LFIA can be reduced by at least one to two orders of magnitude. For example, the first success of enhancing the sensitivity of AuNP-LFIA by using gold growth was achieved by Kaur and co-workers, in which a gold enhancer solution containing HAuCl_4_ and NH_2_OH·HCl was designed and prepared for AuNP enlargement 54. Subsequently, the gold growth enhancement for LFIA has also been demonstrated in detecting other target analytes, including proteins, nucleic acids, and microorganisms 55, 56, 87. Compared with gold growth, silver growth exhibits stronger signal amplification capacity because of its apparent black color after the deposition of metallic Ag onto the AuNP surface, which enables more intensive contrast against the white background of the nitrocellulose membrane 59, 60, 88, 89. Thus, silver growth strategy has been regarded as an effective method to develop highly sensitive LFIA. Horton et al. pioneered the successful use of silver enhancement to improve LFIA detection with a 1000-fold increase in sensitivity 90. Encouraged by this achievement, many improved LFIA methods by silver amplification have been reported for the sensitive determination of multifarious target analytes, such as proteins, pathogens, small molecules, and metal ions 91-95. Copper growth is another common MISG signal amplification approach that could increase the sensitivity of AuNP-LFIA relative to gold or silver growth. Recently, Li et al. explored the use of a copper growth-assisted strategy to enhance AuNP-LFIA for the sensitive detection of human chorionic gonadotropin with the detection sensitivity of 1 pg mL^-1^, which increased about two to three orders of magnitude than conventional AuNP-LFIA without signal amplification 61.

**Figure 5:**
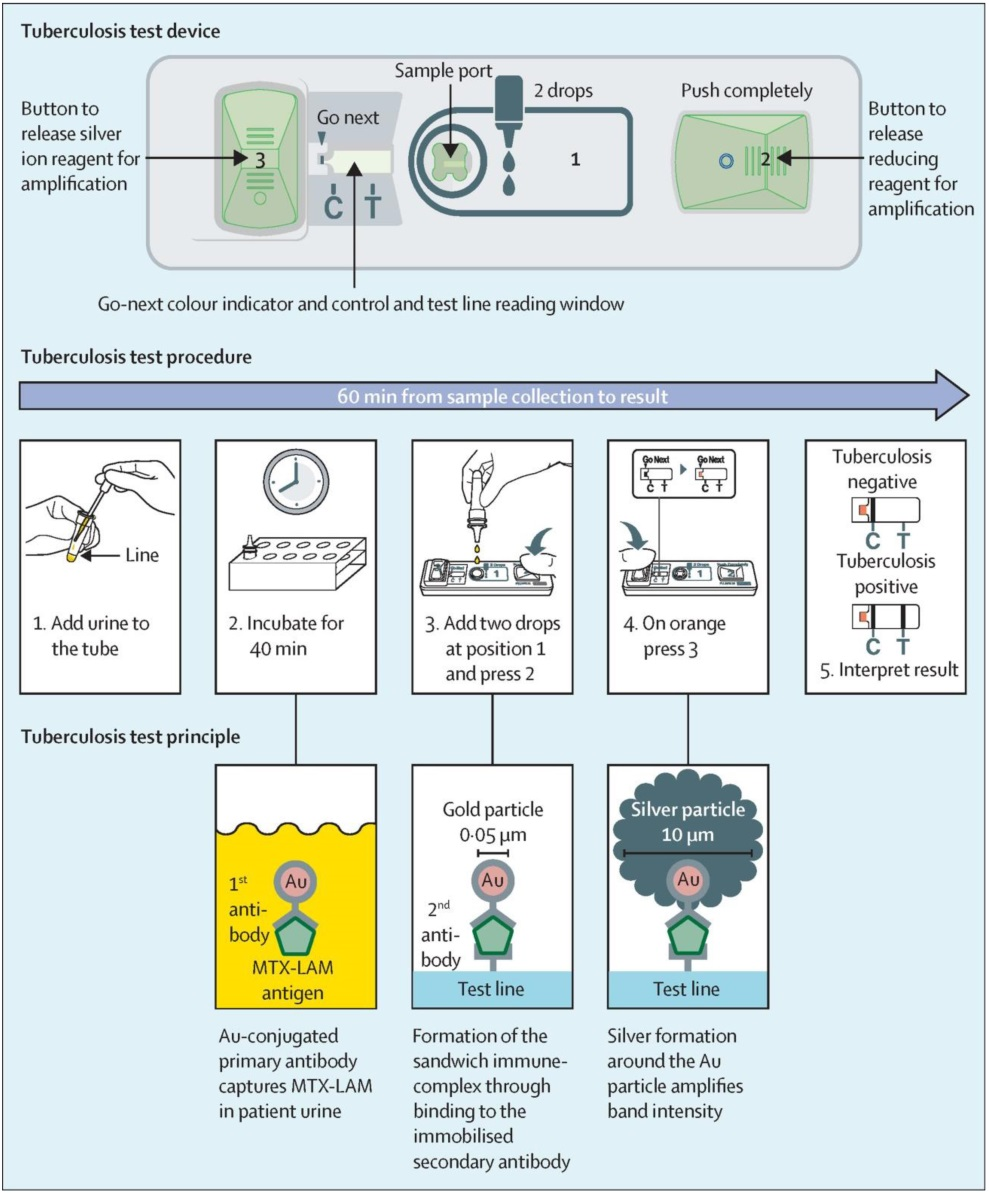
The principle, procedure, and test device of Fujifilm SILVAMP TB LAM. Adapted with permission from 96. Copyright 2020 Elsevier.

Although traditional MISG technologies have obtained a significant progress in improving LFIA detection, their wide applications are still compromised by several limitations. First, the signal amplification ability of the conventional MISG method is limited because it can only conduct one-step growth enhancement. Hence, our group first reported a two-step cascade signal amplification strategy on the conventional AuNP-LFIA platform to achieve ultrasensitive detection of *Escherichia coli* O157:H7 (**Figure 4B**). In this work, a cascade signal amplification strategy consists of gold *in situ* growth and nanozyme-mediated catalytic deposition, which benefits from the ultrahigh peroxidase-mimicking activity of enlarged AuNPs after gold growth. After executing this cascade signal amplification strategy, the enhanced method can detect ultralow concentrations of *E. coli* O157:H7 with a LOD of 1.25 × 10^1^ CFU mL^-1^, which is about 10-fold lower than that of gold growth-amplified strip 58. Second, the traditional MISG strategy primarily relies on a layer-by-layer growth pattern in which the metal shell is deposited directly onto the AuNP surface, thereby resulting in strong background signal and low growth reproducibility caused by the strong nonspecific binding between metal ions and proteins and/or nitrocellulose membrane, self-nucleation of metal ions under excessive reducing agents, and uncontrollable metal growth. Our group has recently developed a polymer-type SDA-assisted controlled copper *in situ* growth strategy to amplify the detection signal of traditional AuNP-LFIA and minimize the background signal caused by nonspecific binding, self-nucleation, and uncontrolled growth (**Figure 4C**). In this design, the controllable signal amplification is achieved by using polyethyleneimine as a SDA to regulate the thermodynamics of anisotropic copper growth on the AuNP surface and restrict free nucleation and reduction of copper ions through stabilizing the chemical potential of the reduction agent and decreasing the free ion concentration. Consequently, the sensitivities of this amplification for p24 antigen and *E. coli* O157:H7 are as low as 50 fg mL^-1^ for p24 antigen and 6 CFU mL^-1^ for *E. coli* O157:H7, respectively 62. Third, the traditional MISG strategy usually needs additional steps of washing and preparing growth solution. Increasing endeavors have been devoted to constructing various modified MISG-based LFIA strips to simplify growth and amplification. For example, a commercially available silver-enhanced LFIA test device (**Figure 5**), namely, Fujifilm SILVAMP TB LAM (FujiLAM), is recently developed by Fujifilm Healthcare for monitoring the presence of lipoarabinomannan in urine to assist the sensitive and accurate diagnosis of tuberculosis. Different from the conventional test strip, this modified device contains two additional buttons located at both ends, which are used to store the silver ion reagent and reducing agent. Consequently, silver growth amplification can be easily achieved by completely pushing the two buttons to release the corresponding growth solution. Compared with the AlereLAM assay for the diagnosis of tuberculosis in people with HIV as recommended by the WHO, FujiLAM displays superior diagnostic sensitivity without compromising specificity and simplicity, although an additional signal amplification step is required 96.

## 3. Nanoparticle shape

**Figure 6:**
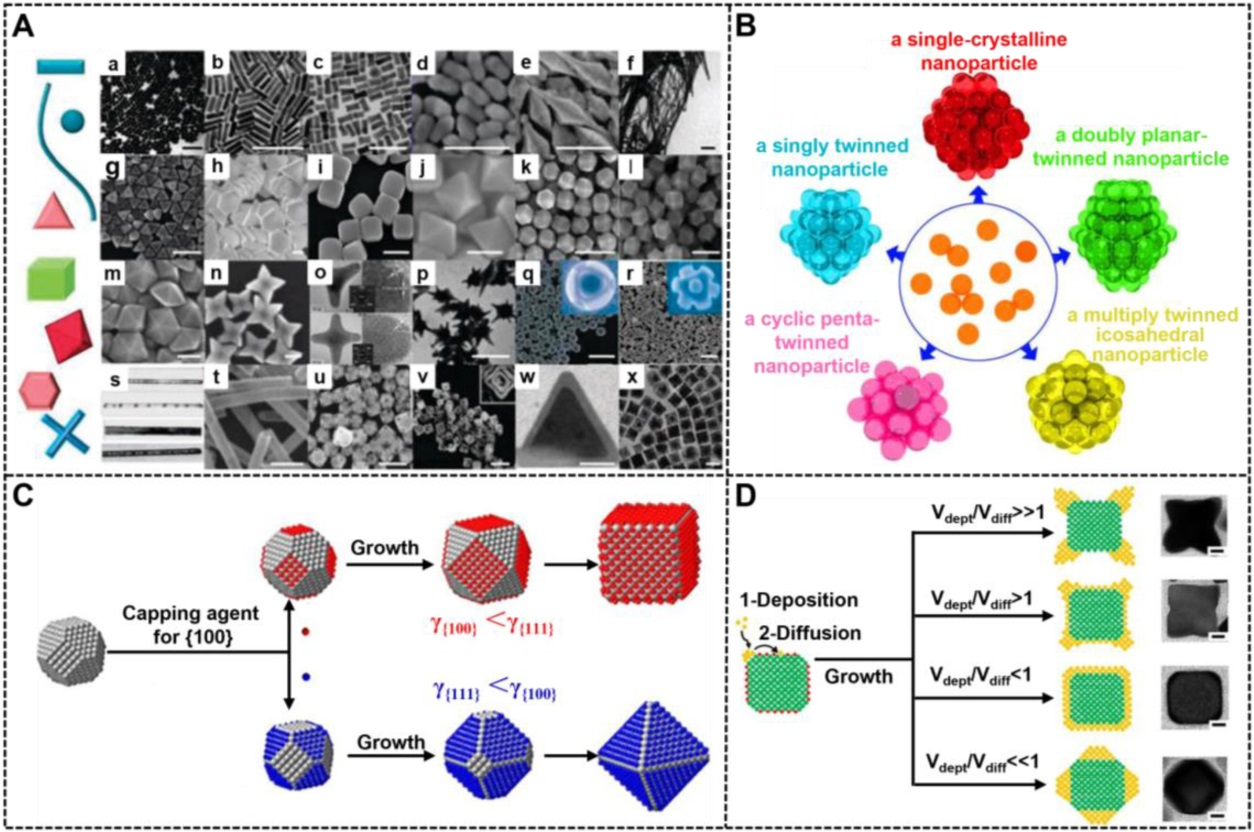
**(A)** Collage of scanning and transmission electron microscopy (SEM and TEM) images of NMNPs with different shapes. Adapted with permission from 97. Copyright 2012 Elsevier. **(B)** Representation of NMNPs with different crystal structures. Adapted with permission from 101. Copyright 2018 American Chemical Society**. (C)** Schematic illustrations showing the role of facet-specific capping agents in controlling the growth pathway of a single-crystal seed made of an *fcc* metal. Adapted with permission from 103. Copyright 2015 American Chemical Society. **(D)** 2D models and TEM images (scale bars: 50 nm) showing the shape evolution of a cubic seed under four different kinetic conditions. The side faces of the cubic seed are covered by capping agents (red dots). Adapted with permission from 27. Copyright 2013 National Academy of Sciences.

In addition to size, the shape of nanoparticles is another important factor affecting their optical properties. NMNPs can be crafted into multifarious desired shapes with fascinating shape-dependent properties. In the last few decades, a myriad of strategies for the synthesis of shape-controlled NMNPs have been reported, and significant advances in this field have been obtained because of sustained contributions. To date, NMNPs are available in a wide variety of shapes (**Figure 6A**) 97, including zero-dimensional (0D) nanospheres; one-dimensional (1D) nanocrystals with circular, square cross-sections (e.g., nanorods and nanowires), and two-dimensional (2D) nanostructures with triangular, hexagonal, or circular projections (e.g., nanoplates, nanosheets, and nanotubes) 98, 99. Compared with spherical nanostructures, these emerging morphologies of NMNPs allow for the fine tuning of their optical properties and functions that are difficult to obtain by simply adjusting the size of spherical nanoparticles. Therefore, the anisotropic growth of NMNPs has been considered as an effective method to modify their optical properties, thereby extending their applications in photocatalysis, optoelectronic devices, biosensing, bioimaging, and nanomedicine with tunable and luxuriant performances. Recently, investigating the influence of shape engineering in NMNPs on the LFIA performance has been enabled by advances in shape-controllable synthesis.

### 3.1. Strategies for nanoparticle shape regulation

Shape control delivers great opportunities for the preparation of various novel NMNPs, thereby increasing their diversities. At present, NMNPs with different shapes are synthesized by two strategies of seed-mediated growth and heterogeneous nucleation. Among them, the seed-mediated growth has aroused wide concern because it has achieved great success in synthesizing different shapes of NMNPs 100. In a typical seed-mediated growth, the shapes of nanoparticles are determined by three important synthetic parameters, including the crystal structures of seeds, the types of capping agents, and the nanocrystal growth kinetics. Controlling the seed crystallinity is prerequisite for high production of NMNPs with the desired shapes. Recently, the role of the seed shape on the structures of final nanocrystals has been systematically summarized by Xia et al 101. **Figure 6B** illustrates the relationships between the seed crystallinity in single crystal; singly twinned, multiply twinned, and plate-like nanostructures; and nanocrystals of face-center cubic metals with different morphologies. Consequently, the recognition of the crystallinity of initial seeds can predict and direct the shapes of the resultant nanostructures. For example, single-crystal seeds can grow into cubes, octahedrons, tetrahedrons, octagonal rods, rectangular bars, and rectangular or octagonal wires; singly twinned seeds can evolve into right bipyramids and beams; multiply twinned seeds can generate icosahedrons, decahedrons, and pentagonal rods, and plate-like seeds can evolve into triangular and hexagonal nanoplates. The internal crystal structures of NMNPs are primarily determined by the seeds, whereas their external crystal structures primarily depend on the growth rate of different seeds' facets. Facet-blocking strategy and growth kinetics control are the two effective approaches for controlling the shape of NMNPs. The facet-blocking strategy relies on the facet-specific capping agents, such as polymers, surfactants, small molecules, and ions, to selectively absorb onto a specific crystal facet of seeds to stabilize the surface atoms and reduce the surface energy (γ) for lowered growth rates, thereby controlling the shapes of resultant NMNPs 102. **Figure 6C** exhibits the role of capping agents in directing the growth of a single-crystal seed into nanocrystals with different shapes, wherein the (111) and (100) planes of a face-centered-cubic (*fcc*) crystal can be selectively blocked and passivated by the corresponding capping agents, thereby inducing seed growth along another unbound directions 103. A detailed discussion of the role of surface capping agents in the shape-controlled synthesis of noble metal nanocrystals is recently reviewed elsewhere. Another common strategy for crystal plane control is to control the growth dynamics of different crystal facets by varying reaction conditions, including reaction temperature, reactant species and concentrations, and reactant adding rate. Consequently, manipulating the deposition rates of atoms and surface diffusion on different planes can selectively facilitate the formation of certain facet, thereby controlling the shape development during seed growth (**Figure 6D**) 27. Compared with the facet-blocking strategy, the kinetic control strategy is more advantageous in the preparation of unfavorable thermodynamic shapes of NMNPs.

### 3.2. Effect of nanoparticle shape on optical properties

**Figure 7:**
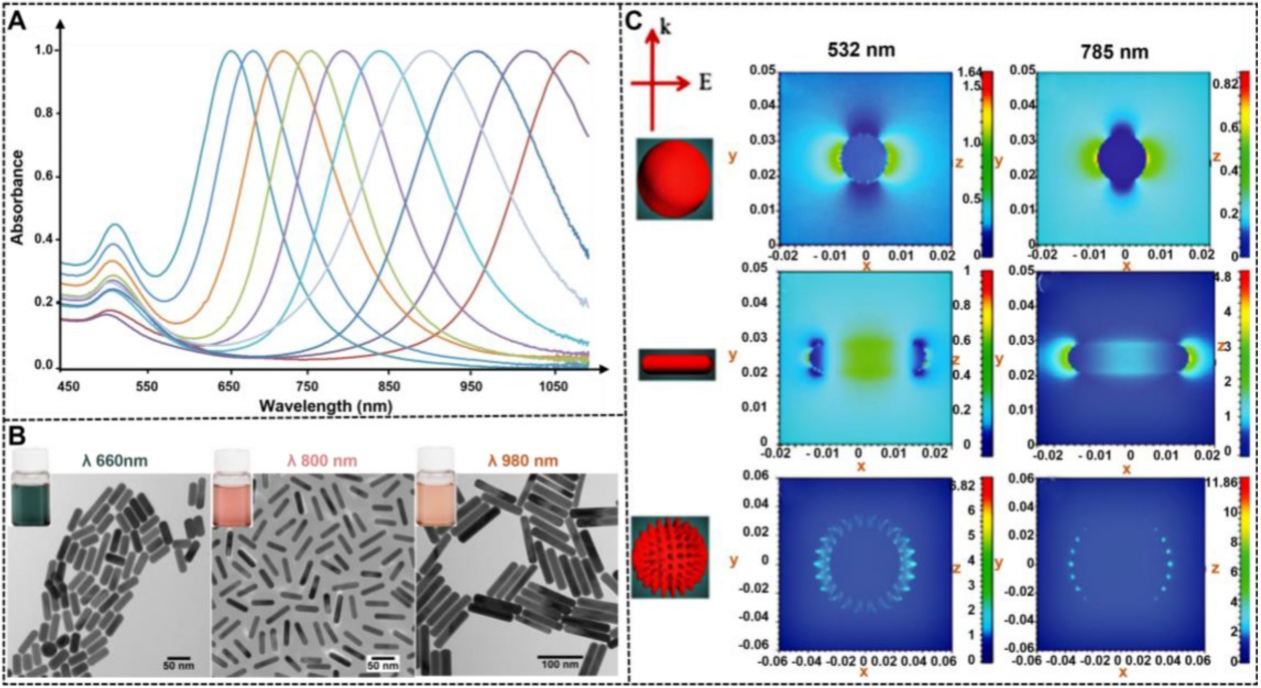
UV-vis spectra **(A)** and TEM images **(B)** of Au nanorods with different aspect ratios. Adapted with permission from 104. Copyright Fortis Life Sciences Company. **(C)** Electromagnetic field distributions and intensities for anisotropic NMNPs (e.g., nanorods and nanostars) compared to spherical NMNPs. The results indicating that the highest near field is generated at the LSPR wavelength and focused on the sharp tips of anisotropic NMNPs. Adapted with permission from 105. Copyright 2017 the Royal Society of Chemistry.

The ability to control the optical properties of spherical NMNPs by the size of nanoparticles is limited, particularly when attempting to extend the LSPR maximum in the near infrared region. In comparison to size, the LSPR of NMNPs is sensitive to their shape variations. As mentioned earlier, the maximal LSPR peak of 50 nm silver nanospheres is located at 450 nm. When the nanospheres are remodeled with nanorods with long and short axes of 50 and 20 nm, respectively, their longitudinal LSPR peaks will be shifted to ∼600 nm. Consequently, the color of the solution changes from yellow to blue. Similar phenomena are observed when spherical AuNPs are reshaped to gold nanorods (AuNRs) with the same size. These observations indicate that the limitation to manipulate the LSPR of NMNPs by size can be easily surmounted by the nanocrystal shape as an important consideration when designing NMNPs, which is due to the fact that anisotropic NMNPs such as rods, triangular prisms, and cubes display multiple LSPRs originated from their reduced shape symmetry. A typical example is gold and silver nanorods, which exhibit tunable longitudinal and transverse LSPRs that are determined by their aspect ratios (**Figures 7A-B**) 104. Moreover, reducing shape symmetry endows locations where the electric field strength is highly concentrated, namely, “hot spots” (**Figure 7C**) 105, thereby promoting highly sensitive detection by SERS. These unique shape-dependent optical properties of MNNPs are stimulating extensive and in-depth investigations in developing strategies to control their shape and expanding their application to a wider range. Collectively, the flexibility in controlling the NMNP shape largely increases the range of LSPR regulation, enriches the color of colloid solution, and improves the electromagnetic field effect, thereby laying a solid foundation for their optical manipulation and sensing applications.

### 3.3. Shape engineering in LFIA

Given the diversities of shape-controlled NMNPs, numerous research groups have explored the use of various anisotropic NMNPs as LFIA reporters to improve the sensitivity. These available anisotropic NMNPs primarily involve multi-branched nanostructures (e.g., nanoflowers, nanostars, and nanopopcorns), hollow nanostructures or nanocages, nanorods, and nanourchins 106-108. Compared with nanospheres, these nanostructures with anomalous morphologies show complex three-dimensional structures and enhanced physicochemical properties, such as good colloidal stability, high colorimetric signal intensity, strong electromagnetic field effects, and excellent photothermal conversion efficiency, thereby favoring the analytical performance of LFIA. Considering such superiorities, significant and tremendous progress in this aspect has been achieved in recent years. In future research, we will review the latest research achievements of the application of shape-controlled NMNPs in enhancing LFIA detection (**Table 3**).

#### Multi-branched nanostructures

**Figure 8:**
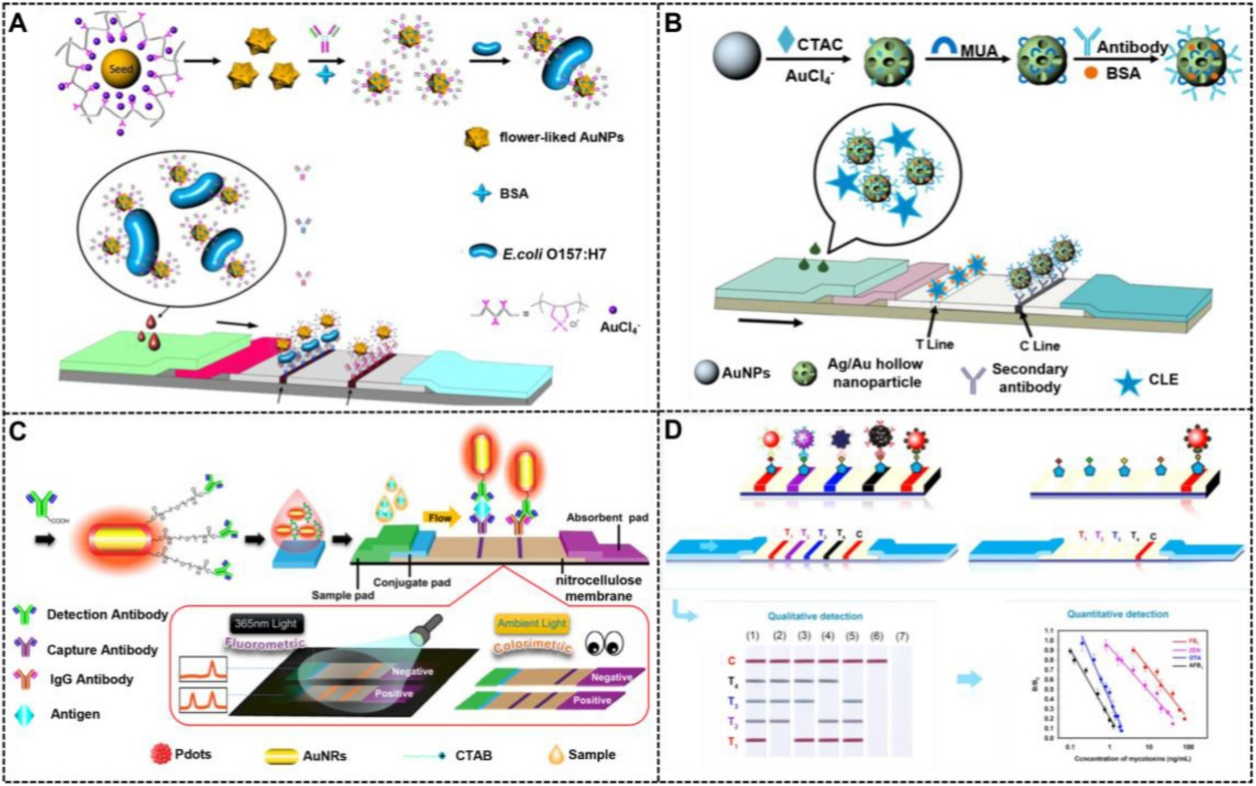
** (A)** Schematic illustration of surface functionalization of flowerlike AuNPs and their LFIA application for detection of *E. coli* O157:H7. Adapted with permission from 35. Copyright 2015 American Chemical Society. **(B)** Schematic illustration of the synthesis and surface modification of hollow Au-Ag NPs and detection of CLE. Adapted with permission from 114. Copyright 2017 Nature-Springer. **(C)** Schematic illustration of preparing colorimetric and fluorescent dual-mode LFIA. Adapted with permission from 120. Copyright 2019 American Chemical Society. **(D)** Multiplex LFIA based shape-controlled nanoparticle for simultaneously detection of fumonisin B1 (FB_1_), aflatoxin B1 (AFB_1_), ochratoxin A (OTA), and zearalenone (ZEN). Adapted with permission from 123. Copyright 2020 Elsevier.

Compared with spherical AuNPs with the same size, multi-branched AuNPs exhibit higher optical signal sensitivity, which benefits from their tips and core-tip interactions that can serve as an antenna to yield electromagnetic field enhancements. In addition, multi-branched AuNPs reveal better colloid stability and larger surface-to-volume ratio than those of spherical AuNPs because of their relatively rough and complex surface 34, 109. Moreover, larger specific surface areas of multi-branched AuNPs are more favorable for improving antibody immobilization than spherical AuNPs with low dimension and ordinary shapes. Such advantages of hierarchical AuNPs make them well suitable as alternative labels of conventional spherical AuNPs with sizes of 20-40 nm to remarkably improve the detection performance of LFIA. For example, our group prepared multi-branched gold nanoflowers (AuNFs) for ultrasensitive and quantitative detection of aflatoxin B1 (AFB_1_) in combination with the competitive LFIA method. The half maximal inhibitory of this method for AFB_1_ at 4.17 pg mL^-1^ was 10-fold lower than the traditional LFIA systems 110. In the same year, Zhang et al. synthesized three hierarchical flower-like AuNPs, namely, tipped flower-like, popcorn-like, and large flower-like AuNPs, and performed a comparative study using these flower-like AuNPs labeled as probes for sandwich LFIA (**Figure 8A**) 35. The results indicate that the tipped flower-like AuNPs exhibit the highest sensitivity of 10^3^ CFU mL^-1^ in testing *E. coli* O157:H7 among the three probes. Subsequently, our group further illustrated the role of the tip length and nanoparticle size in enhancing the performance of competitive and sandwich LFIA 111, 112. In addition, the unique characteristics of multi-branched AuNPs, including multi-branches and surface roughness, result in high SERS activity, which can also increase the LFIA sensitivity. Using 4-aminothiophenol as a Raman reporter molecule and influenza A nucleoprotein as a model analyte, Maneeprakorn et al. demonstrated that the combination of SERS and LFIA could address the low sensitivity of traditional LFIA 113.

#### Hollow nanostructures or nanocages

Except for flower-like nanoparticles, AuNPs with hollow structures were another glorious morphology with high specific surface area for increasing the strong surface affinity with antibodies and plasmonic activities for improving the LFIA sensitivity. Recently, Wang and co-workers optimized the synthesis of bimetallic hollow Ag/Au nanoparticles and investigated their potentials in improving the competitive detection of clenbuterol on the LFIA platform (**Figure 8B**) 114. Under the optimal conditions, the LFIA method could detect low-concentration target with sensitivity at 2 ng mL^-1^, which is superior to that of the conventional LFIA strip using spherical AuNPs as labels. Gold nanocages, as an emerging gold nanomaterial, have received considerable attention because of their special structures with eight vertices and sharp corners/edges. These structural characteristics cause them to serve as “hot spots” and be sensitive to the bulk and local dielectric changes. Given these excellent physicochemical properties, gold nanocages have a wide range of applications in optical sensing and biomedical fields. By combining a sandwich LFIA technology, Yang et al. proved that gold nanocages could serve as enhanced tags for the development of highly sensitive biosensors 115.

#### Nanorods

Compared with spherical AuNPs, AuNRs show more advantages in optical properties, catalytic activity, stability, and biocompatibility. Based on previous reports, the extinction coefficient of AuNRs (~10^9^ L mol^-1^ cm^-1^) is ~10-fold higher than that of spherical AuNPs (~10^8^ L mol^-1^ cm^-1^) 116. Given the anisotropic shape, AuNRs exhibit two distinct LSPR peaks that correspond to the electron oscillations along the transverse and longitudinal directions. Therefore, by adjusting the aspect ratios of AuNRs, they can present different LSPR peaks or rich color variations, thereby providing a favorable basis for rapid screening of visualization using the LFIA method. In addition, unlike nanospheres, nanorods have enhanced electric fields at the tips and edges, which can achieve various enhanced optical properties, such as SERS activity, photothermal effect, and metal-enhanced fluorescence effect, thereby aiding other signal transduction models in LFIA, such as Raman, thermal contrast, and fluorescence. Given these outstanding properties, AuNRs have been widely used for photoacoustic imaging, photothermal therapy, and biosensing 117. The fabrication and application of monodispersed AuNRs as signal reporters in LFIA were first reported by Venkataramasubramani and Tang in 2009 118. Afterward, most of the studies focused on investigating the optimization and use of AuNRs in improving the multiplexing and sensitivity of LFIA, two major challenges encountered by the conventional LFIA. Recently, using gold-silver core-shell bimetallic nanorods as a SERS substrate and 5,5′-dithiobis (2-nitrobenzoic acid) as a Raman reporter, Deng et al. developed a SERS-coupled LFIA strip for ultrasensitive detection of Sudan I with a sensitivity of 0.2 pg mL^-1^ 119. Similarly, He et al. developed a color/SERS dual-mode LFIA method for ultrasensitive detection of *Campylobacter jejuni* by using platinum-coated AuNRs (AuNR@Pt) as signal amplifiers. In this design, AuNR@Pt possesses intrinsic peroxidase-mimicking activities and SERS enhancement properties, thereby facilitating dual-signal readout of color and SERS 120. Under the optimal conditions, the sensitivities of the fabricated dual-mode LFIA are as low as 75 CFU mL^-1^ for the colorimetric mode and 50 CFU mL^-1^ for the SERS mode. By incorporating the fluorescence enhancement effect of AuNRs derived from the electromagnetic coupling with gold surface plasmons, You et al. designed semiconducting polymer dots (Pdots) coated with AuNRs (Au@Pdot) hybrid nanocomposites and demonstrated their uses as a colorimetric/fluorescent dual-modal signal probe in LFIA (**Figure 8C**). Given the plasmon-enhanced fluorescence of Pdots on AuNRs, this developed LFIA shows lower LOD of 1.07 pg mL^-1^ for prostate specific antigen (PSA), which is at least two orders of magnitude better than that of the conventional fluorescent LFIA system. In addition, this dual-modal sensing technology in LFIA application can ensure detection accuracy and reliability 179.

#### Multiplexed LFIA based on shape-controlled NMNPs

Simultaneous detection of multiple target analytes has been a primary development direction in the LFIA area because of the increased detection efficiency and decreased test costs. The exciting progress of multiplexed LFIA has been achieved by integrating the multiline design with monochromatic signal labels. However, this multiplexed LFIA design can hardly provide indicators of line positioning, thereby resulting in the false interpretation of the results when multiple successive bands with the same color are present in a small test area 121. By contrast, multicolored signal labels can effectively avoid these risks and enable intuitive visualization because each color corresponds to an analyte in a single strip. In recent years, increasing attempts have been devoted to constructing various multicolored LFIA strips by using different colored reporters as alternatives to traditional monochromatic ones. In this content, the shape or size-controlled NMNPs with different distinct colors are desirable signal probes for the development of multicolored LFIA. Emulating this principle, several multicolored LFIA strips were successfully developed for the determination of various target analytes, including proteins, mycotoxins, and viruses. Yen and co-workers presented a multiplexed LFIA design for multiplexed disease diagnostics using multicolored silver nanoparticles as labelling probes. In their work, three different silver nanoparticles with distinguishable colors (orange, red, and green) were synthesized using a seed-mediated growth strategy to control the morphological evolution. After coupling with antibodies against dengue virus NS1 protein, Yellow Fever Virus NS1 protein, and Ebola virus Zaire strain glycoprotein, this design successfully achieves multiplexed determination of the three pathogens 122. Similarly, our group explored the first application of gold nanospheres (red), gold nanocacti (purple), AuNFs (blue), and hyperbranched Au plasmonic blackbodies (black) in a multiplexed LFIA for the rapid and simultaneous detection of four common mycotoxins (**Figure 8D**) 123.

## 4. Nanoparticle elemental composition

In general, the noble metals can be divided into two categories of coinage metals (e.g., Au and Ag) and platinum-group metals (e.g., Pd, Pt, Rh, Ir, and Ru) 124. Noble metal nanomaterials consisting of coinage metals show tunable LSPR properties, but they have limited catalytic activities. By contrast, the nanostructures originated from platinum-group metals exhibit exactly opposite properties 27. NMNPs with a single component can hardly meet the requirements with respect to high optical sensitivity, excellent colloid stability, and high catalytic activity. In addition, a small change in elemental composition of noble metal nanostructures can make a huge difference 124. Accordingly, nanomaterial fabrication has gradually shifted toward the design and development of composite NMNPs containing two or more noble metal components or one noble metal incorporating non-noble metals with different physicochemical properties 40, 125. Composite nanomaterials effectively combine the functions of each component while avoiding its limitations, thereby significantly improving their overall properties, such as tunable optical properties 126, increased colloid stability 127, and enhanced catalytic activities 36, and emerging new synergistic properties, such as magnetic 128 or fluorescent properties 39. In recent years, composite noble metal nanostructures with controlled dimensions and tunable morphologies have been synthesized using various methods. Given their superior performances and remarkable synthesis, composite NMNPs have obtained numerous applications in a wide range of areas, including catalysis, optoelectronics, biomedical devices, sensing, and imaging 37. As an improved signal reporter for LFIA, the composite NMNPs have been demonstrated with enhanced detection performances relative to traditional LFIA using single-component NMNPs as labels. Therefore, the following discussion will highlight the current state of the art of controlled synthetic strategies and performance improvements of composite noble metal nanostructures and their applications in LFIA. **Table 4** summarized the representative LFIAs using elemental composition-controlled NMNPs.

### 4.1. Strategies for nanoparticle elemental composition regulation

Given their advantages over corresponding monometallic nanoparticles, composite noble metal nanomaterials are particularly useful for diverse applications. At present, the synthesis of composite nanomaterials with core-shell, alloy, and bimetallic heterostructures has been achieved via various approaches, including atomic layer deposition, electrochemical deposition, templated growth, chemical reduction, thermal decomposition, sonochemical method, and biosynthesis 124. Among these methods, chemical reduction is the commonly used method. The procedure for synthesizing composite nanomaterials using this method is similar to that of monometallic nanoparticles, however controlling the co-reduction of mixed metal precursors is more challenging and complicated owing to their different reduction potential and inherent chemical properties. In general, the simultaneous reduction of mixed metal precursors in a homogeneous solution will produce alloyed nanocomposites, whereas a sequential reduction can lead to core-shell nanostructured composites 129. Therefore, a series of synthetic parameters, such as the crystallization of each component, surface energy, relative bonding strength among different elements, relative atomic radius, and surface capping agent, should be considered to obtain the expected composite nanostructures with unexpected properties. In particular, some mild reducing agents, such as ethylene glycol and diethylene glycol, citrate and sodium citrate, ascorbic acid, formic acid, 2-thiopheneacetonitrile, polymers, and oleylamine, can be considered for use in synthesizing monodispersed composite nanomaterials in solution to achieve better control over the reaction kinetics and final product 130. With all this in mind, core-shell nanoparticles or other nanohybrids of noble metals and non-noble metals are among the combinations that have been well studied 131. To date, many composite noble metal nanomaterials, such as core-shell nanostructures (e.g., a metal shell surrounding a gold core or a gold shell surrounding another metal core) and alloyed bimetallic nanostructures (e.g., Au@Pt Co@Pt, Au@Ag, and Pt@Pb), serve as potential alternatives to traditional single-component AuNPs for the establishment of high-performance LFIA 24, 132, 133.

### 4.2. Effect of elemental composition on the properties of composite NMNPs

The elemental composition of composite NMNPs plays a crucial role in their properties, wherein a slight change in the elemental composition can have a significant effect on the performance of composite nanomaterials in enhancing the optical signal intensity, increasing the colloidal stability, and breeding new functionalities 130. Although the commercialized colorimetric signal labels have dominated by spherical AuNPs with sizes of 20-40 nm in LFIA, these citrate-modified AuNPs often suffer from low signal intensity and poor colloidal stability against variations in environmental factors, thereby resulting in low sensitivity and poor resistance to matrix interference in real practice. This issue can be well addressed by introducing polydopamine coating to form ultra-stable core-shell nanostructured composites due to its high chemical reactivity, strong adhesion capacity, good biocompatibility, and excellent stability 134. In another typical example, silver nanostructures show good LSPR activity, but they suffer from poor chemical and structural stability. Thus, increasing the stabilities of silver nanomaterials is the key to their wide applications. An available approach for this goal is through incorporating gold using galvanic replacement to produce Ag-Au nanocomposites 135, 136.

Noble metal nanomaterials with high catalytic activities have attracted extensive attention in enhancing the detection signal intensity of LFIA labels. Such catalytic bimetallic nanomaterials can be obtained by incorporating a catalytic noble metal into another metal within individual nanocomposite using the co-reduction or sequential reduction strategy 36. The resultant multimetallic nanoparticles of different elementals, containing Au@Ag 135, Au@Pt 36, Pt@Pd 133, Pd@Ir 137, and Au@Ag@Pt 138, have been intensively studied in enhancing the colorimetric signal intensity due to their stronger catalytic activities compared with their parent monometallic nanoparticles. The catalytic constants of such noble metal nanozymes reach up to 10^4^-10^5^ s^-1^, about one to two orders of magnitude higher than that of horseradish peroxidase (10^3^ s^-1^) 139. In several recent studies, Xia's group has substantially improved the catalytic efficiencies of noble catalytic nanomaterials with a catalytic constant of 10^6^-10^7^ s^-1^ 36, which could serve as excellent substitutes for developing highly sensitive biosensors. In addition, multifunctional noble metal nanomaterials are emerging composite nanostructures that can simultaneously possess additional properties other than the functionalities of noble metal nanomaterials, which have attracted considerable interest in various fields. These multifunctional composite nanomaterials can be constructed by using the evaporation-induced self-assembly and the post-modification or post-growth method 37, 41. For example, the combination of noble metal compositions and magnetic compositions to form composite magnetic NMNPs exhibits magnetic and plasmonic behavior, which can achieve simultaneous magnetic enrichment and colorimetric sensing 140. Another notable example is the composition of noble metal materials and fluorescent materials, which allow the simultaneous generation or regulation of two signal transduction channels of colorimetry and fluorescence, thereby facilitating a novel dual-modal sensing technology to achieve some synergistic effects on the detection performances 141. In brief, composite NMNPs have advantages in at least two niche fields, which offer an elegant strategy for overcoming the unsolved bottlenecks encountered by single-component nanomaterials.

### 4.3. Elemental composition engineering in LFIA

#### Composite metallic nanozymes

**Figure 9:**
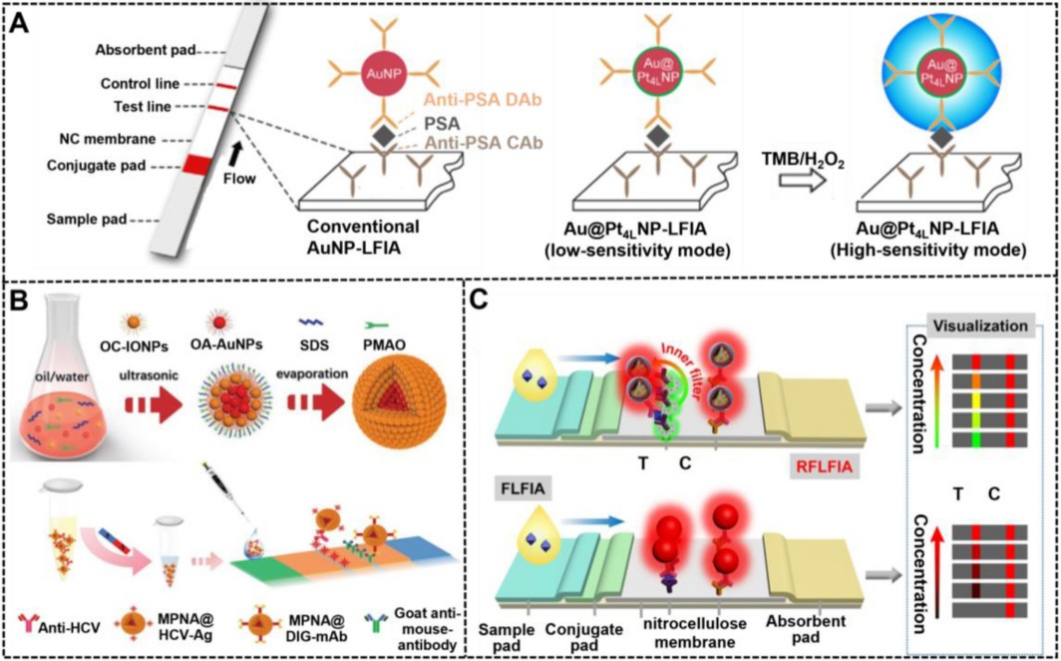
** (A)** The comparison between conventional AuNP-based LFIA and Au@Pt nanoparticle-based LFIA for PSA detection. Adapted with permission from 36. Copyright 2019 American Chemical Society. **(B)** Schematic illustration for the synthetic procedures of magnetic-plasmonic nanoassemblies (MPNAs) and their application in a sandwich LFIA for the diagnosis of hepatitis C virus infection. Adapted with permission from 37. Copyright 2019 Wiley-VCH GmbH. **(C)** Illustration of RFLFIA and traditional FLFIA for visual and quantitative detection of H-FABP. Adapted with permission from 38. Copyright 2021 Wiley-VCH GmbH.

Different from traditional colored nanomaterials, catalytic NMNPs exhibit dual roles for generating colorimetric signal transduction by intrinsic plasmonic activity and performing enzymatic deposition amplification by peroxidase-like activities. Leveraging these nanomaterials as signal probes for LFIA, about one order of magnitude improvement in sensitivity has been achieved compared with conventional LFIA without amplification 139, 142. However, this enhancement is limited by the relatively low catalytic efficiency of monometallic nanozymes. As described earlier, composite metallic nanozymes have stronger catalytic efficiency than monometallic nanomaterials with similar sizes, which can further improve the detection sensitivity of LFIA when used as reporters. Recently, several multimetallic nanohybrids, such as Au@Pt 24, Pt@Pd 133, Pt@Ni 143, and Au@Ag@Pt 138, have improved the analytical performance of LFIA. For example, Gao et al. synthesized a series of difunctional Au@Pt_nL_ (nL: n atomic layers of Pt) nanohybrids through engineering traditional ~40 nm AuNPs with ultrathin Pt coating of 1-10 atomic layers by varying the amount of Pt precursor during seed growth and then studied the impact of Pt shell thickness on their catalytic activity 36. The results suggested that the catalytic efficiency of Au@Pt_nL_ increased sharply with the increase of atomic layers and then reached a constant at n = 4. Using the synthesized Au@Pt_4L_ nanohybrids as signal labels, the developed LFIA could achieve an ultrasensitive detection for PSA with an LOD of 20 pg mL^-1^ in combination with Au@Pt_4L_-mediated enzymatic deposition amplification, which presents two orders of magnitude enhancement in sensitivity relative to that of Au@Pt_4L_-based LFIA without catalytic amplification (**Figure 9A**). Around the same time, Loynachan et al. reported a similar porous core-shell Au@Pt nanozyme and demonstrated its potential for improving the POC diagnostics of HIV coupled with the amplification of enzymatic deposition signal on the LFIA platform.

#### Magnetic-plasmonic nanohybrids (MPNHs)

Composite MPNHs are a promising multifunctional nanomaterial because of their intrinsic plasmonic and magnetic properties, which support their various uses, such as magnetic separation and optical sensing. High-performance MPNHs should retain high magnetic-plasmonic activities simultaneously, thereby promoting fast magnetic response and sensitive colorimetric signal transduction. At present, most of MPNHs are prepared by growing Au shell or attaching isolated Au AuNPs onto the surface of magnetic nanomaterials to form core-shell “gold-coated magnetic” nanostructures 144. However, given the inherent magnetic shielding effect of Au shell on the surface of magnetic nanomaterials, the saturation magnetization of such core-shell nanostructures decrease significantly with the increase of the plasmonic component. Although the shielding effect can be efficiently weakened via introducing a spacer layer between magnetic and plasmonic units, such as silicon and PDA layer, the synthesis of high-quality MPNHs with the maximized saturation magnetization and plasmonic activity remains a challenge. 125 In recent research, our group reported a facile synthesis of an improved MPNH, namely, MPNAs, through co-assembling oleylamine-coated AuNPs (OA-AuNPs) with oleic acid-coated iron oxide nanoparticles (OC-IONPs) into polymer nanospheres (**Figure 9B**) 37. The resultant MPNAs show a typical “magnetic-coated gold” core-shell nanostructure with OA-AuNPs aggregating into a core and OC-IONPs assembling into a magnetic shell, thereby achieving an efficient spatial separation of OA-AuNPs and OC-IONPs to minimize the interference between them. Thanks to the high loading of OA-AuNPs and reasonable spatial distribution of OC-IONPs, the obtained MPNAs were characterized with high saturation magnetization (~79.5% of initial OC-IONPs) and dramatically enhanced plasmonic activities. After covalently conjugating with recognition elements against hepatitis C virus antibody, the MPNAs can achieve synchronous magnetic enrichment and colorimetric sensing of target analytes on the magnetic-assisted LFIA platform based on their intrinsic dual functionality of plasmonic and magnetic. Under the optimized conditions, the LOD of this method was 0.24 pg mL^-1^, which was below those obtained by other control groups.

#### Fluorescent-plasmonic nanohybrids (FPNHs)

Composite FPNHs are another multifunctional nanoprobe, which are extensively studied because of their intrinsic dual-mode signal transduction of plasmon and fluorescence 145. Recently, several typical FPNHs, such as AuNPs/AgNPs-organic fluorophore-based and AuNPs/AgNPs-quantum dot (QD) nanocomposite, have been reported for use in bioassay, photocatalysis, and imaging 40. The available synthetic approaches for FPNHs primarily include the incorporation of plasmonic and fluorescent nanoparticle by layer-by-layer polyelectrolyte coating, the direct linkage of two nanomaterials using a chemical strategy, or the emulsion-based self-assembly technology 146, 147. However, fluorescence may be quenched in the nanocomposite due to the plasmon-induced resonance energy transfer of plasmonic nanomaterials. The precise control of the distance between plasmonic units and fluorescent units and their spatial distribution in individual FPNH are critical to minimize fluorescence quenching and even obtain metal-enhanced fluorescence. Consequently, Huang et al. reported a synthetic method for high-quality FPNHs, namely, SASQS, by using the dendritic silica template to assemble directly with hydrophobic AuNPs via thiol-metal coordination. After silanization, controlled silica growth, and mercapto-group grafting, hydrophobic red-light-emitting QDs were then immobilized densely to form the nanocomposite of SASQS. Through further functionalization of antibodies against cystatin C (Cys C), this SASQS can perform the dual-mode colorimetric and fluorescent sensing of Cys C with LODs of 0.61 and 0.24 ng mL^-1^, respectively 40. Subsequently, by integrating green-light-emitting QDs (denoted as gQDs) as a reference fluorescent signal, a ratiometric fluorescent LFIA strategy, namely, RFLFIA, was described by the same group for highly sensitive quantitative detection of heart-type fatty-acid-binding protein (H-FABP) integrated with a smartphone (**Figure 9C**) 38. In this work, the SASQS conjugated with the detected antibody (SASQS-mAb1) was sprayed in the conjugate pad, whereas gQDs modified with the captured antibody (gQD-mAb2) were immobilized at the test line. Upon the addition of H-FABP, the sandwich immunocomplex was formed, thereby triggering the fluorescent change from green to yellow and then to red. This phenomenon may be due to the overlap of red fluorescence from SASQS and green fluorescence from gQDs and the inner filter effect of AuNPs from SASQS on the green emission. Compared with traditional FLFIA with only monochromatic fluorescent change, the proposed RFLFIA strip is more distinguishable due to its green-to-red color variance.

## 5. Nanoparticle external structure

The enhancement in the optical, photothermal, or catalytic properties of NMNPs largely depends on their external structure. NMNPs with unique external structures can create plasmonic coupling for an intense electromagnetic field within the confined area, thereby significantly modifying the optical properties of NMNPs or other optical molecules adjacent to their surface 148. Consequently, increasing researchers aimed to manipulate the optical activities of NMNPs, such as plasmonic, SERS, and fluorescent signal, to improve their application performance 27. In addition to controlled nanocrystal synthesis, the optical properties of NMNPs can be enhanced by other methods. The LSPR property of NMNPs is closely associated with interparticle coupling interactions. Considering the LSPR, a significant enhancement in light absorption and local electromagnetic field is observed with the decrease of the interparticle distance. Thus, several high-quality plasmonic NMNPs with specific nanostructures of nanoaggregate 82, core-shell 149, and core-satellite characteristics 150 have shown potential applications in diverse areas, including biosensing, of such carefully crafted plasmonic nanostructures for enhancing the LFIA performance have been well investigated. **Table 5** summarized some representative LFIAs based on external structure-controlled NMNPs.

### 5.1. Strategies for the construction of structure-directed nanoparticles

Synthesizing NMNPs with related structures is crucial to achieve the fine tune of their optical properties and functions, which has motivated researchers to develop strategies for controlled preparation of such specific nanostructured NMNPs. For example, nanoaggregates can be prepared via chemical coupling and nucleic acid hybridization. For example, Hu et al. reported the simple synthesis of oligonucleotide-linked AuNP nanoaggregates by modifying a pair of complementary oligonucleotide sequences onto the surface of spherical AuNPs to form corresponding conjugates as crosslinking precursors 151. Compared with individual AuNPs, AuNP nanoaggregates exhibit enhanced colorimetric signal strength, which is attributed to the collective molar extinctions of AuNPs. Nevertheless, the synthetic controllability and reproducibility of such methods are relatively poor. In addressing this issue, the controllable assembly strategy was recently reported to improve the synthesis of nanoaggregates and nanoassemblies. Recently, our group reported that assembled colloidal gold superparticles (abbreviated as GSPs) with remarkably increased light absorption and improved colloid stability were synthesized by the self-assembly of small hydrophobic AuNPs into large GSPs 82.

Another effective approach to amplify the optical signal intensity to yield visual enhancement of NMNPs is through the design of core-satellite composite nanoarchitectures, wherein the core-satellite nanocomposite can be synthesized by attaching a large amount of isolated NMNPs onto the surface of large nanocarriers via the *in-situ* growth of NMNPs, and the post-modification of NMNPs, such as physical absorption and covalent coupling. To date, the available nanocarriers primarily include NMNPs, magnetic nanomaterials 152, 153, silica nanorods (SiNRs) 154, graphite-like carbon nitrides 155, latex nanocomposites 150, metal organic frameworks 156, and bacteria 157. In addition, the core-shell feature of NMNPs benefits the SERS enhancement and thus allowing the SERS biosensing owing to the electronic ligand effect and enhanced electromagnetic field located in core-shell nanostructures, that can cause evident Raman signal enhancement for Raman reporter molecules deposited between interfaces. These core-shell NMNPs are usually synthesized by the sequential reduction growth method. Currently, the SERS activities of Ag@Au, Au@Ag, Au@Ag@Au, and hollow Au-Ag core-shell nanoparticles were investigated 149, 158. In addition, several studies have attempted to elucidate the effect of gap size between the core and the shell on their SERS signal enhancement performance, and the results indicated that the gap size should match the expected thickness of the monolayer of Raman reporter molecules 159. The coupling of NMNP-based SERS tags and LFIA technologies improves the sensitivity and quantification, thereby increasing its actual application value in POC testing. Hence, the following section will summarize the typical applications such structure-directed NMNPs in improving the LFIA.

### 5.2. Structure engineering in LFIA

#### Nanoaggregates and nanoassemblies

**Figure 10:**
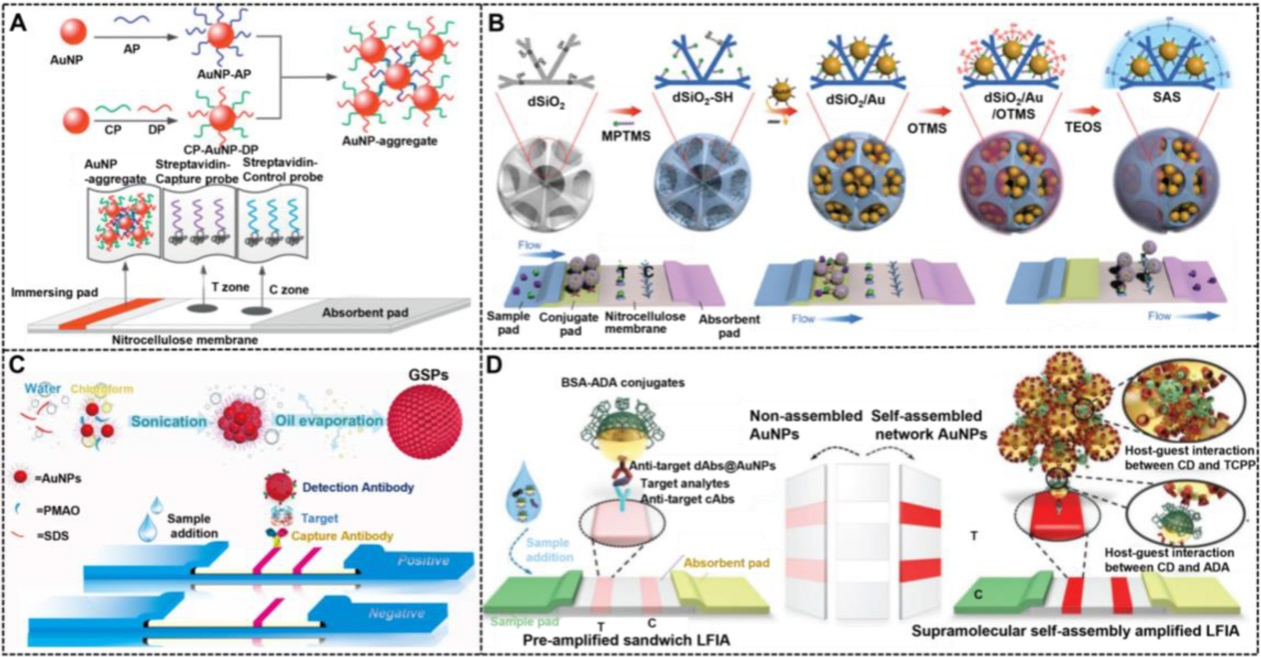
** (A)** The preparation of oligonucleotide linked AuNP aggregates and design of improved LFIA. Adapted with permission from 151. Copyright 2013 the Royal Society of Chemistry. **(B)** Schematic illustration for the preparation of AuNP implanted nanospheres and their application in LFIA. Adapted with permission from 161. Copyright 2019 the Royal Society of Chemistry. **(C)** Illustration of the synthetic strategy for GSPs by the microemulsion-based self-assembly process and their application in LFIA. Adapted with permission from 82. Copyright 2020 Theranostics. **(D)** Schematic representation of the design and fabrication of supramolecular self-assembly amplified LFIA. Adapted with permission from 162. Copyright 2019 Wiley-VCH GmbH.

Increasing the accumulation of AuNPs on the T line has been considered as the straightforward strategy for enhancing the sensitivity of traditional AuNP-LFIA. Xu and co-workers used oligonucleotide-linked AuNP nanoaggregates as amplified signal probes to fabricate an improved LFIA for sensitive detection of a nucleic acid sequence of HIV (**Figure 10A**) 151. The LOD of this developed lateral flow assay was 0.1 nM, thereby providing a 2.5-fold enhancement in detection sensitivity compared with that of traditional strip without signal amplification. Taking advantage of the immunoreaction to assist the synthesis of AuNP nanoaggregates for amplifying the plasmonic signal, Rivas et al. recently reported an enhanced LFIA methodology for the improved detection of *Leishmania* parasite DNA with a LOD of 0.038 parasites in each DNA amplification reaction 160. AuNP nanoassemblies consisting of numerous isolated AuNPs reveal markedly enhanced intensity, thereby increasing the LFIA sensitivity. Huang et al. recently reported the preparation of a controllable and high-quality nanoassembly, namely, SAS, by implanting high-density hydrophobic AuNPs into three-dimensional silica scaffolds (**Figure 10B**) 161. The resultant SAS nanoassemblies were further functionalized with antibodies and used for highly sensitive POC testing of methamphetamine with a LOD of 0.026 ng mL^-1^ coupled with the LFIA. Similarly, our group demonstrated the improved analytical performances of assembled AuNP nanomaterials (GSPs) with remarkably enhanced light absorption in monitoring proteins and pathogens (**Figure 10C**) 82, which resulted from the high loading capacity and weak interparticle plasmonic coupling in the nanoassemblies. Nevertheless, the signal amplification abilities of these methods are often compromised by the limited accumulation of nanoparticles at the T line, which only provide about one order of magnitude increase in sensitivity. Recently, our group developed a supramolecular self-assembly mediated multiple rounds of signal amplification strategy for enhancing the sensitivity of conventional AuNP-LFIA (**Figure 10D**) 162. In this design, the smart host-guest supramolecular recognition between β-cyclodextrin (β-CD)-coated AuNPs and adamantane (ADA) or tetrakis(4-carboxyphenyl) porphyrin (TCPP) was applied to achieve the layer-by-layer self-assembly and massive accumulation of AuNPs at the T line. Under the optimized conditions, the amplified LFIA exhibited ultrahigh sensitivity with a LOD at the subattogram level and super-wide detection range covering seven orders of magnitude. In addition, the method can customize biomarker detection to meet the needs of clinicians by using a controllable cycle self-assembly strategy.

#### Core-satellite nanostructures

**Figure 11:**
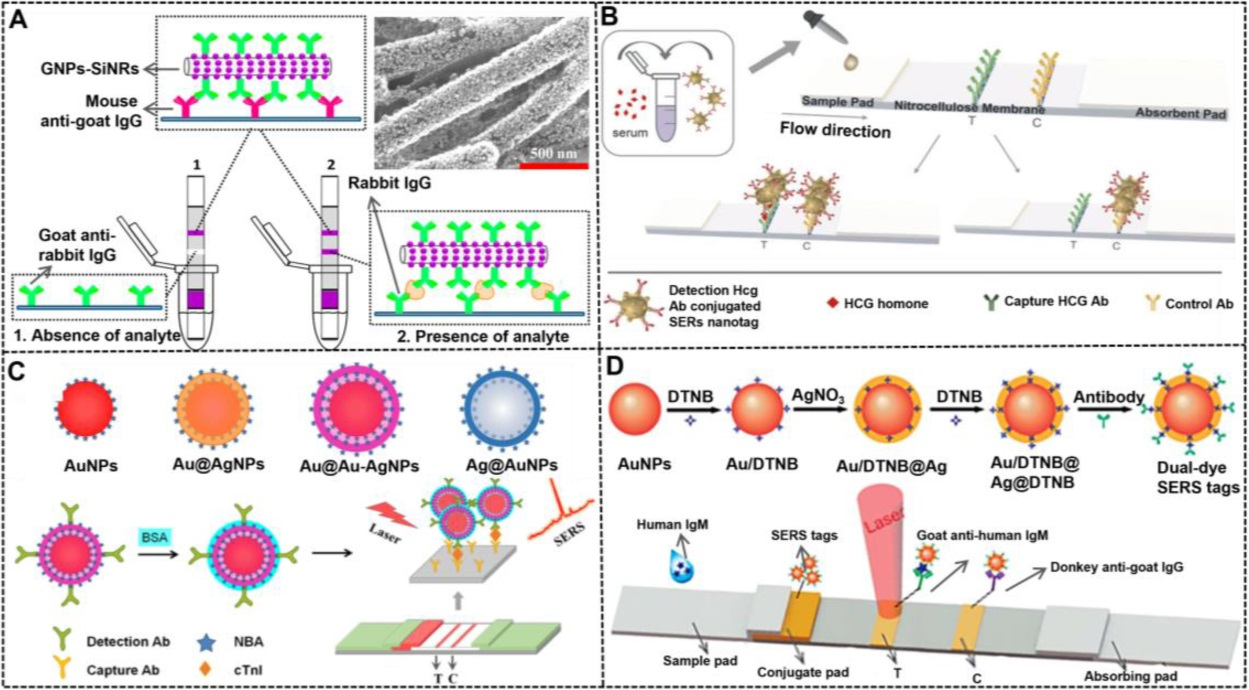
** (A)** Schematic representation for the configuration and the measurement principle of the LFIA based on AuNPs loaded SiNR; Adapted with permission from 154. Copyright 2014 American Chemical Society. **(B)** Schematic representation for the SERS-labeled Au/Ag satellites probes for detection of HCG in real serum sample. Adapted with permission from 166. Copyright 2018 Wiley-VCH. **(C)** The structural schematic diagram of four different types of nanoprobes with core-shell structures, and the principle of the SERS-based LFIA for detection of cardiac troponin I (cTnI). Adapted with permission from 158. Copyright 2018 Anal Bioanal Chem. **(D)** Synthetic route for dual dye-loaded SERS tags and schematic illustration of quantitative detection of human IgM using SERS-based LFIA. Adapted with permission from 176. Copyright 2018 the Royal Society of Chemistry.

Composite core-satellite nanostructured nanoparticles containing large numbers of AuNPs on their surface can produce intensive color signals when compared with pure AuNPs, thereby increasing the detection sensitivity of traditional AuNP-LFIA. Xu et al. pioneered the synthesis of AuNP-decorated SiNRs (AuNP-SiNRs) with enhanced colorimetric signal intensity and then used them as colored LFIA probes to improve protein detection (**Figure 11A**) 154. In their work, AuNP-SiNRs were synthesized by using a two-step deposition process, including gold seed deposition and its growth. As shown in the inset of **Figure 11A**, a layer of AuNPs with a density at around 10^4^ was deposited on the surface of a single SiNR, thereby resulting in a purple color that was darker than pure AuNP solution. After being modified with antibodies, the AuNP-SiNRs can measure rabbit IgG at the concentration of 0.01 ng mL^-1^. Consequently, increasing scholars have focused on the construction of various core-satellite nanostructures for the enhanced determination of other target analytes, involving aflatoxin B_2_ 153, 157, human chorionic gonadotropin (HCG) 156, 163, microRNA 164, clenbuterol, 17β-estradiol 155, CRP 165, and influenza A antigen 150 with or without additional signal amplification.

Except for providing enhanced colorimetric signal transduction, the composite core-satellite nanostructures can also serve as improved SERS nanoprobes owing to their strong Raman signal enhancement efficiency originated from the formed “hot spots” between core and satellites. Tran et al. developed an Au/Au core-satellite nanocomposite as a SERS nanotag (**Figure 11B**) 166, which consisted of a 50 nm spherical AuNP core and 17 nm AuNP satellites. This nanocomposite was prepared through electrostatic assembly between the positively charged core and negatively charged satellites, wherein the core was pre-modified with the Raman molecule thio-2-napthol and bridging molecule (11-mercaptoundecyl)-N,N,N-trimethyl-ammonium. Given the strong plasmonic coupling between core and satellites, many hot spots are generated, and the Raman signal of reporter molecules in multiple narrow gaps is significantly enhanced. After the electrostatic adsorption of antibodies onto its surface, this Au@Au core-satellite nanoprobe can successfully measure HCG at low concentrations of 1.6 mIU mL^-1^ by combining with a home-made portable Raman strip reader.

#### Core-shell nanostructures

Previous studies have indicated that the use of SERS signals to replace colorimetric signals of AuNPs is a potential strategy to produce high sensitivity of at least two orders of magnitude increase. The structure of SERS nanotags plays an important role in the LFIA. Plasmonic core-shell nanostructures have attracted great interest attributed to their unique tunable LSPR property, which have been widely adopted for the design and development of SERS nanoprobes 167, 168. Compared with other nanostructures, the core-shell structure displays many inherent advantages. First, the Raman reporter molecules can be optionally placed at the surface of nanoparticles or embedded in the gap between the core and the shell, or both. Second, the outer metal shell layer can serve as not only a signal amplifier, but also a protective coating to prevent the leakage and degradation of Raman reporter molecules. Third, the multi-layer Raman molecules, multi-shelled nanostructures, and gap size between the core and the shell in a core-shell nanocomposite are adjustable, following the requirement of the actual application. These characteristics make core-shell nanostructures an ideal option for manufacturing high-performance SERS nanotags. Since its first discovery in 2007, core-shell SERS nanoprobes have attracted considerable attention and extensive research 169. Given the excellent stability and monodispersity of gold and high enhancement effect of silver, AgNPs@Au-shell 170, AuNPs@Au-shell 171, and AuNPs@Ag-shell 172 SERS nanoprobes have been reported. Blanco-Covián et al. synthesized AuNPs@Ag-shell with 34 nm AuNPs as a core coated with a 12 nm silver shell by using a seed-mediated growth process 132. The resulting AuNPs@Ag-shell was then modified with the Raman reporter to prepare the SERS nanoprobes. Using this nanoprobe as a signal label, the LOD of this SERS-enhanced LFIA was 1 pg mL^-1^ for pneumolysin. As an improvement, Li et al. synthesized AuNPs@Ag-shell SERS nanoprobes (Au^MBA^@Ag) by embedding the Raman reporters (4-mercaptobenzoic acid, MBA) in the junction between the core and the shell, which can effectively protect the Raman molecules from the interference of environmental factors and possible leakage and degradation 173. Subsequently, several similar AuNPs@Ag-shell SERS nanotags have been developed for the improved LFIA detection of salbutamol 169 and bacteria 174. In addition, the metal shell number of core-shell nanostructures is increased to enhance the SERS performance. Shen et al. designed AuNPs@Ag-shell@Ag-shell SERS nanoprobes (AuAg^4-ATP^@AgNPs) as a highly sensitive signal to enable high sensitivity in the LFIA 175. This enhanced SERS performance by increasing the metal shell was further confirmed by a recent comparative study from Wang's group, in which four different SERS nanoprobes, namely, AuNPs, AuNPs@Ag-shell, AgNPs@Au-shell, and AuNPs@Ag-shell@Au-shell, were comprehensively investigated by theoretical analysis and experimental measurement (**Figure 11C**). The results verified the advantage of AuNPs@Ag-shell@Au-shell in the following aspects, including SERS activity, simulated electromagnetic field, and LFIA sensitivity 158.

Apart from AuNPs@Ag-shell, AgNPs@Au-shell exhibits high sensitivity in SERS-based LFIA due to the higher enhancement effect of AgNPs than AuNPs. Zhang et al. synthesized AgNPs@Au-shell core-shell bimetallic SERS nanotags (AgNBA@Au) with the Raman reporter (methylene blue and Nile blue A) distributed in the interior gap between the two metals 170. By optimizing the thickness of Au shell, the obtained core-shell SERS nanotags with the highest intensity were used as labelled probes to improve the multiplex testing of cardiac biomarkers and respiratory tract infection pathogens on the lateral flow test strip. The introduction of multilayer Raman reporter molecules in the core-shell nanostructures has also been reported with improved sensitivity. Jia et al. used AgNPs@Au-shell as a carrier to load two layers of Raman dye 5,50-dithiobis-(2-nitrobenzoic acid) (DTNB) on the surface and in the interior gap to prepare SERS nanotags (Au^DTNB^@Ag^DTNB^, **Figure 11D**) 176. Raman spectrum analysis indicates that Au^DTNB^@Ag^DTNB^ shows 1.5- and twofold higher SERS signal than those of Au^DTNB^@Ag and Au@Ag^DTNB^, respectively. The LOD of this SERS nanotag for human IgM was 0.1 ng mL^-1^, which was 100-fold more sensitive than the colorimetric strategy.

## 6. Summary and outlook

Conventional colorimetric LFIA using 20-40 nm spherical AuNP as labels has suboptimal sensitivity compared with laboratory-based immunoassays. The growing demands in highly sensitive detection of analytes have necessitated the improvements of the LFIA sensitivity. Various approaches focusing on five major core elements involved in the LFIA system, including sample, receptor, interaction, response, and signal output, have been enabled to reach lower LOD and higher sensitivity. Thanks to their excellent physicochemical properties, NMNPs have presented a wide range of applications in improving LFIA. In particular, the properties of NMNPs could be precisely controlled by tightly tuning their physicochemical parameters, including size, shape, composition, and external structure. Compared with traditional spherical AuNPs, the engineered NMNPs exhibit a series of enhanced performances in optical properties, colloid stabilities, catalytic activities, and multifunctional manipulations, thereby favoring the development of high-performance LFIA. Therefore, in this review, we discussed recently developed strategies using engineered NMNPs with desired properties to improve the sensitivity of LFIA. Despite the incredible progress made in this area, many scientific issues and technical challenges must be addressed prior to applications.

One of the challenges is the large-scale controlled synthesis of high-quality and high-performance engineered NMNPs. Achieving the high-yield and scaling-up synthesis protocol for industrial use is important. The uniformity of nanoparticles in size, shape, composition, and structure has been achieved using the existing synthesis technologies. However, the synthesis robustness and reproducibility of the currently available strategies are too low to meet the requirement of industrial implementations. Therefore, further efforts should be devoted in developing new synthetic methods that allow nanoparticle products to be highly reproducible and controllable in yield, morphology, composition, and purity.

The analytical performance of NMNPs in LFIA is determined by their properties. In addition, the properties of NMNPs are closely associated with their physicochemical parameters. Thus, the precise control of the physicochemical parameters of NMNPs is crucial to obtain the optimal performance in LFIA applications. Benefiting from the rapid advances in nanoparticle synthesis and characterization, noble metal nanomaterials with controllable structure parameters and charming physicochemical properties have been obtained. Nevertheless, more advanced engineering methods and characterization tools should be introduced. Moreover, theoretical, and mechanistic studies on NMNP engineering should be conducted, thereby contributing to the understanding of spatio-temporal evolution variation of nanoparticles for the control of their structural design. Although several studies have focused on the relationships among the physical parameters of NMNPs, their physicochemical properties, and LFIA performances, further investigations of the correlations among these three factors are greatly encouraged, which can promote the application of the LFIA.

Noble metal nanomaterials with catalytic activities are regarded as potential alternatives to traditional AuNPs in the LFIA because they provide dual functions of plasmonic colorimetric sensing and nanozyme-catalyzed amplification. The catalytic activities of such nanomaterials are closely related to their composition, shape, size, and surface functionalization. For example, the nanozyme activities of catalytic nanomaterials will markedly decrease with surface modification of various ligands as compared with naked nanoparticles. Notably, surface functionalization of nanoparticles with recognition elements (e.g., antibodies) is the premise to ensure the stability and selectivity of labels in LFIA. Thus, the appropriate surface coating must be considered to retain the catalytic activities of nanomaterials and ensure good performance in LFIA. In addition, other structural parameters that affect the enzyme-mimicking activities of nanomaterials, such as size and shape, must be explored to ensure the superior performance of LFIA. Furthermore, the design and synthesis of novel noble metal nanostructures with ultrahigh catalytic activity and the exploration of their potential as catalytic tags for LFIA are good options.

Metal growth amplification is an important strategy for significantly enhancing the colorimetric signal intensity to enable lower LOD in LFIA. However, most of the current strategies are uncontrollable and limited by the self-nucleating growth of metal ions, thereby resulting in high background signal and low reproducibility, and greatly reducing the accuracy and reliability of methodologies. In this regard, controllable growth techniques with improved robustness are necessary to broaden its application in practice. Analogous to the enzymatic deposition amplification, the metal growth amplification is also a post-amplification technique that is widely used to enhance the colorimetric signal strength after completing the strip. However, these two signal amplification strategies rely on two separate steps: running the strip with a sample solution and treating the completed strip with an enhancer solution, thereby leading to large batch-to-batch differences between strips. Therefore, the ingenious integration of these two independent operations into an entire strip device can reduce the variability and improve the reproducibility. For example, the combination of LFIA using a microfluidic technology can achieve simultaneous detection and signal amplification of samples in a single test system by controlling the direction of flow of different liquids.

Incorporating engineered NMNPs with other functional nanomaterials to increase functionalities has attracted increasing interest in developing various noble metal-based multifunctional composite nanostructures. Given their intrinsic multifunctionality, these hybrid nanomaterials have exhibited superior properties over their parent nanomaterials, thereby endowing traditional LFIA with additional performances, such as magnetic manipulation to separate and enrich analytes from the complex sample and multimodal sensing to improve the analytical efficiency and performance and increase the flexibility of application. Although various approaches have been successfully applied to synthesize these composite nanomaterials, the preparation of high-performance multifunctional nanohybrids remains a challenge, which might be due to the interaction of noble metal components and other functional components. For example, the noble metal nanomaterials can quench the emission of fluorescent materials by the inner filter effect and plasmon-induced resonance energy transfer. Interestingly, when the distance between the noble metal components and fluorescent component is appropriate, a metal-enhanced fluorescence effect can be observed. Therefore, during the synthesis of composite nanomaterials, we should focus on minimizing the adverse interferences and maximizing the beneficial effects between noble metal components and other components.

Currently, the signal readout modes for noble metal nanomaterials mainly involve the qualitative screening by naked eyes and the quantitative analysis by the dedicated strip readers with multiple signal transduction modes, like fluorescence, photothermal, and SERS signals. Compared with the qualitative screening modes, these alternative signal-reading methods usually rely on the use of strip readers, which can provide better analytical performances and quantitative measurements, but increase the operational difficulty, testing complexity, and cost. Therefore, the exploitation of less sophisticated reader technology to allow the quantitative analysis in LFIA is highly desired.

In summary, this paper systematically reviews the role of noble metal nanoparticle label design in enhancing the LFIA sensitivity. However, it should be mentioned that in addition to the nanoparticle design, other important factors influencing the LFIA sensitivity should be considered including the screening of more efficient recognition molecules, the bioactivities of antibodies coupled onto the surface of NMNPs, the membrane selection (*e.g.*, aperture), the anti-fouling sensing ability, the sample pretreatment modification, the migration behavior of nanoparticles, the development of signal amplification strategies, and the strip device engineering. Several relevant research papers that focus on the above perspectives to enable sensitive or even ultrasensitive LFIA test strips have been authorized recently 43, 46, 47, 197-203. Eventually, we hope that with these challenges well addressed, more engineered noble metal nanostructures can earn their place in LFIA applications in addition to traditional AuNPs.

## Figures and Tables

**Table 1:** A summary of some representative LFIAs by size-controlled noble metal nanoparticles.

Size range of AuNPs	Optimal size	Analyte	LOD	Ref.
20-39 nm	39 nm	Progesterone	No given	85
40-150nm	80 nm	DNA	50 fmol	177
10-40 nm	20 nm	Prostate acid phosphatase	0.25 ng mL^-1^	84
6.4-52 nm	33.4 nm	Potato virus X	3 ng mL^-1^	86
14-38 nm	38 nm	Hepatitis B surface antigen	No given	178
34-137.8 nm	42.7 nm	Hepatitis B surface antigen	10 ng mL^-1^	179
20-180 nm	100 nm	Ochratoxin A	0.27 ng mL^-1^ (IC_50_)	53
30-100 nm	100 nm	C-reactive Protein	1.6 pM	33
16-115 nm	115 nm	Cardiac troponin I	0.03 ng mL^-1^	32

**Table 2:** A summary of some representative LFIAs based on the metal *in-situ* growth strategy.

Noble metal growth	Analyte	Dynamic range	LOD	Ref.
Gold growth	Atrazine	No given	1 ng mL^-1^	54
	HCG	No given	0.3 mIU mL^-1^	55
	DNA of *E. coli* O157:H7	0.39-3.13 nM	0.4 nM	56
	*E. coli* O157:H7	5×10^3^-1.6×10^5^ CFU mL^-1^	5×10^3^ CFU mL^-1^	87
	*S. enteritidis*	10^3^-10^8^ CFU mL^-1^	10^4^ CFU mL^-1^	58
	*E. coli* O157:H7	1.25×10^2^-2.5×10^5^CFU mL^-1^	12.5 CFU mL^-1^	57
Silver growth	Cardiac troponin I	No given	0.016 ng mL^-1^	59
	Abrin-a	0.1-100 ng mL^-1^	0.1 ng mL^-1^	60
	H5N5	0.5-250 ng mL^-1^	0.5 ng mL^-1^	88
	Avian influenza virus	2^1^-2^-9^ dilution	2^-12^ dilution	89
	Newcastle disease virus	No given	2^-10^ dilution	
	Fumonisin B_1_	No given	2 ng mL^-1^	91
	*Ralstonia solanacearum*	2×10^3^-2×10^6^ CFU mL^-1^	2×10^2^ CFU mL^-1^	92
	Prostate specific antigen	No given	0.1 ng mL^-1^	93
	Potato leafroll virus	No given	0.2 ng mL^-1^	94
	Troponin I	No given	0.24 ng mL^-1^	95
Copper growth	HCG	1 pg mL^-1^-100 ng mL^-1^	1 pg mL^-1^	61
	p24 antigen	50 fg mL^-1^-1 ng mL^-1^	50 fg mL^-1^	62
	*E. coli* O157:H7	No given	6 CFU mL^-1^	

**Table 3:** A summary of some representative LFIAs by engineering the shape of noble metal nanoparticles.

Methods	Signal	Analyte	Dynamic range	LOD	Ref.
Multibranched nanostructures	Colorimetry	Aflatoxin B1	0.5-25 pg mL^-1^	4.17 pg mL^-1^ (IC_50_)	110
	Colorimetry	*E. coli* O157:H7	No given	10^3^ CFU mL^-1^	35
	Colorimetry	OTA	No given	0.61 ng mL^-1^ (IC_50_)	111
	Colorimetry	Influenza A	No given	67 ng mL^-1^	113
	SERS	-nucleoprotein		6.7 ng mL^-1^	
	Colorimetry	HCG	9-2304 mIU mL^-1^	9 mIU mL^-1^	112
	Colorimetry	S-100β	0.1-100 ng mL^-1^	5.0 pg mL^-1^	34
Hollow nanostructures	Colorimetry	Clenbuterol	No given	2 ng mL^-1^	114
	Colorimetry	IgG	0.5-50 ng mL^-1^	0.1 ng mL^-1^	115
Nanorods	LSPR shift	ErbB2 antigen	No given	No given	118
	Colorimetry	*E. coli* O157:H7	10^2^-10^6^ CFU mL^-1^	100 CFU mL^-1^	36
	Colorimetry	*Campylobacter jejuni*	10^2^-10^6^ CFU mL^-1^	75 CFU mL^-1^	120
	SERS	*Campylobacter jejuni*	10^2^-5×10^6^ CFU mL^-1^	50 CFU mL^-1^	42
	Fluorescence	Prostate-specific antigen	3-10 ng mL^-1^	1.07 pg mL^-1^	180
Nanoprisms	Temperature	HCG	35-7000 mIU mL^-1^	2.8 mIU mL^-1^	181
Multiplexed LFIA	Colorimetry (orange, red and green)	Dengue virus	No given	150 ng mL^-1^	122
		Yellow fever virus	No given		
		Ebola virus	No given		
	Colorimetry (blue, yellow, and red)	Ovalbumin	No given	0.1 ng mL^-1^	182
		Hazelnut allergen	No given		
		Casein	No given		
	Colorimetry (red, purple blue, and black)	Fumonisin B_1_	4-80 ng mL^-1^	3.27 ng mL^-1^	123
		Zearalenone	0.8-40 ng mL^-1^	0.70 ng mL^-1^	
		Ochratoxin A	0.2-2 ng mL^-1^	0.10 ng mL^-1^	
		Aflatoxin B_1_	0.1-1.25 ng mL^-1^	0.06 ng mL^-1^	

**Table 4:** A summary of some representative LFIAs using elemental composition-controlled noble metal nanoparticles.

Element	Analyte	Dynamic range	LOD	Ref.
Pt	HCG	0.1-9 ng mL^-1^	0.2 ng mL^-1^	139
	HCG	No given	0.3 ng mL^-1^	183
	Dehydroepiandrosterone	1-1000 ng·mL^-1^	10.0 ng mL^-1^	142
IrO_2_	IgG	No given	0.07 μg mL^-1^	184
	Salbutamol	0.18-12 ng mL^-1^	0.002 ng mL^-1^	127
Au-Ag	Cadmium ion	0.05-25 ng mL^-1^	0.05 ng mL^-1^	126
	Penumolysin	No given	1 pg mL^-1^	132
	Chloramphenicol	1.36-123.21 ng mL^-1^	0.36 ng mL^-1^	172
	Thiamphenicol	0.80-95.29 ng mL^-1^	0.20 ng mL^-1^	
	Florfenicol	2.09-71.53 ng mL^-1^	0.78 ng mL^-1^	
	Prostate‑specific antigen	0.3-10.00 ng mL^-1^	0.20 ng mL^-1^	135
Au-Pt	Prostate‑specific antigen	10-200 pg mL^-1^	20 pg mL^-1^	36
	p24	1-10000 pg mL^-1^	0.8 pg mL^-1^	24
	Rabbit IgG	0.05-10 ng mL^-1^	5 pg mL^-1^	185
	*Clavibacter michiganensis*	No given	300 CFU mL^-1^	186
	Potato virus X	No given	4-8 pg mL^-1^	187
Pt-Ni(OH)_2_	Acetochlor	0.1-20 ng mL^-1^	0.63 ng mL^-1^	143
		1.0-150 ng mL^-1^		
	Fenpropathrin	0.1-20 ng mL^-1^	0.24 ng mL^-1^	143
		1.0-150 ng mL^-1^		
Pt-Pd	p53 Protein	0.1-10 ng mL^-1^	0.05 ng mL^-1^	188
	*E. coli* O157:H7	10-10^7^ CFU mL^-1^	34 CFU mL^-1^	133
	*E. coli* O157:H7	1×10^3^-1×10^6^ CFU mL^-1^	9.0×10^2^ CFU mL^-1^	189
	Butyrylcholinesterase	0.05-6.4 nM	0.025 nM	190
Au-Ag-Pt	Myoglobin	6.67-150 ng mL^-1^	5.47 ng mL^-1^	138
FPNHs	H-FABP	No given	0.21 ng mL^-1^	38
	Dicofol	23.98-478.32 ng mL^-1^ (colorimetry)	9.9 ng mL^-1^ (colorimetry)	39
		3.97-91.47 ng mL^-1^ (fluorescence)	1.59 ng mL^-1^ (fluorescence)	
	Glycoprotein	2-1000 ng mL^-1^	0.18 ng mL^-1^	141
	Cystatin C	0.12-500 ng mL^-1^ (colorimetry)	0.61 ng mL^-1^ (colorimetry)	40
		0.12-500 ng mL^-1^ (fluorescence)	0.24 ng mL^-1^ (fluorescence)	
MPNHs	Anti-HCV	0.24-120 pg mL^-1^	0.24 pg mL^-1^	37
	Treponema pallidum antigens	No given	1 NCU mL^-1^	140
	Single nucleotide polymorphism	5 ng-1200 ng per test	5 ng per test	191
	Single nucleotide polymorphisms	0.02-2 pg μL^-1^	0.04 pg μL^-1^	192
	β-conglutin	10 fM-100 pM	8 fM	144
	H1N1	No given	50 pfu mL^-1^	41
	HAdV	No given	10 pfu mL^-1^	
	HCG	0-50 mIU mL^-1^	0.2 mIU mL^-1^	125
	HCG	0.01-4 mIU mL^-1^	0.0094 mIU mL^-1^	128
	*E. coli* O157:H7	10^2^-10^5^ CFU mL^-1^	9 × 10^1^ CFU mL^-1^	

**Table 5:** A summary of external structure-controlled noble metal nanoparticles based LFIA.

Structure	Analyte	Dynamic range	LOD	Ref.
Core-shell	Phenylethanolamine A	No given	0.32 pg mL^-1^	173
	Salbutamol	No given	3.0 pg mL^-1^	169
	*Salmonella enteritidis*	2.7-2.7×10^6^ CFU mL^-1^	27 CFU mL^-1^	174
	Respiratory infection virus	1 pM-50 nM	0.03-0.041 pM	193
	Pseudorabies virus	41-650 ng mL^-1^	5 ng mL^-1^	175
	Pharmaceutical diclofenac	No given	0.07 pg mL^-1^	194
	Clenbuterol	0-1 ng mL^-1^	5 ng mL^-1^	171
	Myoglobin	0.01-500 ng mL^-1^	3.2 pg mL^-1^	22
	Cardiac troponin I	0.01-50 ng mL^-1^	0.44 pg mL^-1^	
	Creatine kinase-MB isoenzymes	0.02-90 ng mL^-1^	0.55 pg mL^-1^	
	Cardiac troponin I	No given	0.1 ng mL^-1^	149
	Mycoplasma pneumoniae (IgM)	0.1 ng -10 μg mL^-1^	0.1 ng mL^-1^	176
	Mycoplasma pneumoniae	0.1-10 mg mL^-1^	0.1 ng mL^-1^	
	Cardiac troponin I	0.09-50 ng mL^-1^	0.09 ng mL^-1^	158
Core-statellite	Aflatoxin B_2_	No given	0.9 ng mL^-1^	153
	rabbit IgG	0.05-2 ng mL^-1^	0.01 ng mL^-1^	154
	HCG	No given	0.039 mIU mL^-1^	163
	MicroRNA	No given	10 pM	164
	HCG	No given	1.6 mIU mL^-1^	166
	Clenbuterol	0.1-2 ng mL^-1^	0.1 ng mL^-1^	157
	C-reactive protein	No given	0.08 ng mL^-1^	165
	Influenza A	No given	2.5×10^-2^ HAU mL^-1^	150
	17β-estradiol	No given	0.5 ng mL^-1^	155
Nanoaggregates	HIV-1	No given	0.1 nM	151
	Nucleoside	No given	20 µM	195
	p24 antigen	0.1 fg mL^-1^-10 ng mL^-1^	0.01 fg mL^-1^	162
	Methamphetamine	0.023-375 ng mL^-1^	0.026 ng mL^-1^	161
	HCG	0.49-1000 mIU mL^-1^	0.49 mIU mL^-1^	82
	Hepatitis B surface antigen	0.46-1000 ng mL^-1^	0.45 ng mL^-1^	
	Troid-stimulating hormone	0.4-40 μIU mL^-1^	0.5 μIU mL^-1^	196
